# Protein transduction therapy into cochleae via the round window niche in guinea pigs

**DOI:** 10.1038/mtm.2016.55

**Published:** 2016-08-17

**Authors:** Hiroki Takeda, Takaomi Kurioka, Taku Kaitsuka, Kazuhito Tomizawa, Takeshi Matsunobu, Farzana Hakim, Kunio Mizutari, Toru Miwa, Takao Yamada, Momoko Ise, Akihiro Shiotani, Eiji Yumoto, Ryosei Minoda

**Affiliations:** 1 Department of Otolaryngology-Head and Neck Surgery, Kumamoto University, Kumamoto, Japan;; 2 Department of Otolaryngology, National Defense Medical College, Tokorozawa, Japan; 3 Department of Molecular Physiology, Kumamoto University, Kumamoto, Japan

## Abstract

Cell-penetrating peptides (CPPs) are short sequences of amino acids that facilitate the penetration of conjugated cargoes across mammalian cell membranes, and as such, they may provide a safe and effective method for drug delivery to the inner ear. Simple polyarginine peptides have been shown to induce significantly higher cell penetration rates among CPPs. Herein, we show that a peptide consisting of nine arginines (“9R”) effectively delivered enhanced green fluorescent protein (EGFP) into guinea pig cochleae via the round window niche without causing any deterioration in auditory function. A second application, 24 hours after the first, prolonged the presence of EGFP. To assess the feasibility of protein transduction using 9R-CPPs via the round window, we used “X-linked inhibitor of apoptosis protein” (XIAP) bonded to a 9R peptide (XIAP-9R). XIAP-9R treatment prior to acoustic trauma significantly reduced putative hearing loss and the number of apoptotic hair cells loss in the cochleae. Thus, the topical application of molecules fused to 9R-CPPs may be a simple and promising strategy for treating inner ear diseases.

## Introduction

**Figure 1:**
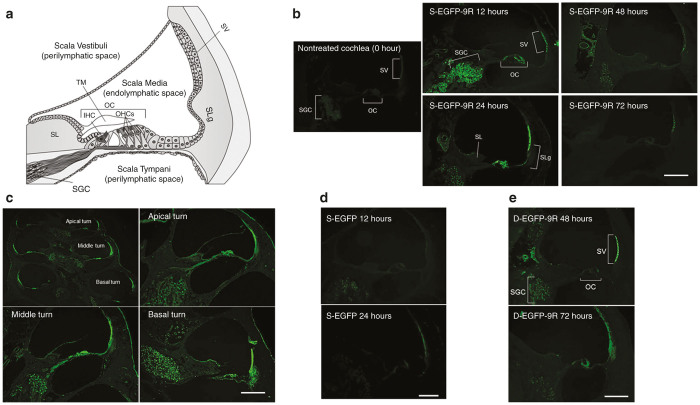
Sectional images after enhanced green fluorescent protein (EGFP) or EGFP-9R transduction. (**a**) Cross-sectional diagram of a normal adult cochlea. The adult mammalian cochlea consists of three compartments: the scala vestibuli; scala tympani; and, scala media. The scala media contains the organ of Corti (OC). The OC contains inner hair cells (IHCs) and outer hair cells (OHCs). The IHCs and outer hair cells (OHCs) act as mechano-electrical transducers and play a crucial role in hearing. The electrical signal is transmitted via the spiral ganglion cells (SGC) to the auditory pathway of the brain. The stria vascularis (SV), located in the lateral wall of the scala media, is responsible for the secretion of K^+^ into the endolymph and the production of the endocochlear potential. (**b**) The images show sections of the middle turns of the cochleae. In the s-EGFP-9R group, EGFP was detectable strongly in the SV, OC and SGC at 12 hours. Also EGFP was detectable strongly in the SV, OC, and SGC, and slightly in the spiral limbus (SL) and the spiral ligament (SLg) 24 hours. EGFP levels were moderately detectable at the SV and SG at 48 hours and only slightly detectable at the SV at 72 hours. (**c**) At 24 hours in the s-EGFP-9R Group. EGFP was detectable at all turns in the cochlea. (**d**) At the middle turn of the cochleae in the s-EGFP group, EGFP was slightly detectable in the SGC at 12 hours and in the SV at 24 hours. (**e**) At the middle turn of the cochleae in the d-EGFP-9R group, EGFP was significantly detectable in the SV and SGC at 48 hours. The scale bars indicate 50 μm.

Treatment modalities for human sensorineural hearing loss have not yet been fully established. A major obstacle for this is a lack of an effective and safe delivery method for therapeutic drugs or molecules into cochleae, because mammalian inner ears have unique and complex anatomical structures: a perilymphatic space; and, an endolymphatic space where both auditory and vestibular sensory cells exist. The cochlea of the inner ear, which contains auditory sensory cells, is one of the most critical structures in the auditory pathway. The cochlea consists of three fluid filled compartments: the scala tympani; the scala vestibule; and the scala media (Figure 1a). The surface of the hearing sensory organ is covered by endolymph that fills the scala media. The inner ear is isolated from the systemic circulation by the “blood-labyrinth barrier”,^1^ and from the middle ear by the round window membrane (RWM). These complex structures make drug delivery into the inner ear difficult.^2^ Given this background, there are two routes to achieve inner ear drug delivery that are used clinically in humans: the systemic route and the intratympanic route. Systemic administration has been used clinically for glucocorticoid treatments of acute sensorineural hearing loss and Meniere’s disease. Intratympanic administration though appears to be superior to systemic administration. Systemic administration tends to require higher-dosage regimens because of the blood–labyrinth barrier and the extent of the systemic circulation. Besides such high-dose regimens can cause undesirable side effects.^3–5^ Contrarily, intratympanic drug administration may be able to avoid the adverse systemic effects thus generating therapeutic effects in the inner ear via the RWM which consists of three layers: an outer epithelium; a middle core of connective tissue; and an inner epithelium.^6^ The RWM is the membranous septum between the middle ear and the perilymphatic space in the cochleae in humans, monkeys, felines, and rodents. Because of the limited permeability nature of the RWM the development of treatment delivery strategies may be challenging. Permeability through the RWM can be affected by factors such as molecular size, configuration, concentration, liposolubility, electrical charge level, and membrane thickness.^7,8^ Regarding size, *in vivo* experiments have revealed that 1-μm microspheres can traverse chinchilla RWMs, whereas 3-μm spheres cannot.^9^ Lundman *et al*.^10^ demonstrated that the passage of endotoxins with a molecular weight >100,000 through a normal RWM was limited. Thus, lower molecular weight molecules pass through the RWM, while higher molecular weight molecules do not readily pass through the RWM. Indeed, intratympanic drug therapy utilizing lower molecular weight molecules has been performed successfully clinically, as is shown in the two following examples: aminoglycoside antibiotics (*e.g.*, gentamicin: its molecular weight is 478) for the treatment of Meniere’s disease; and glucocorticoids (*e.g.*, dexamethasone: its molecular weight is 392) for the treatment of sensorineural hearing loss.^7^ Although the impenetrable nature of the RWM for higher molecular weight molecules may be a defense system for the inner ear, the lack of penetration by higher molecular weight molecules could simultaneously constitute a barrier to good therapeutic outcomes when intratympanic drug therapy is applied via the RWM for inner ear dysfunction.

The human immunodeficiency virus type 1 trans-activator of transcription (TAT) protein can enter cells upon addition to a culture medium.^11,12^ The transduction of TAT, which contains a high proportion of arginine and lysine residues, is responsible for the ability of the virus to be able to penetrate the plasma membrane.^13^ Short peptide sequences, such as the protein transduction domain of TAT, are referred to as “cell-penetrating peptides” (CPPs). CPPs are short cationic peptides with the ability to traverse the cell membranes of several types of mammalian cells.^13–15^ Various macromolecules have been attached to these peptides and subsequently internalized. The cargo molecules that penetrate the cell membranes maintain their biological activities.^13–15^ CPPs are generally used for protein transduction and RNA-based gene silencing to interrupt or suppress gene expression.^16^ Among the various CPPs, arginine-rich peptides have been the most widely studied.^12,14,17–20^ Simple polyarginine peptides, with an optimal length of 9–11 residues, induce significantly higher cell penetration rates than TAT proteins do.^19–22^ Considering this superior nature of polyarginine peptides, attachment of polyarginine peptides to higher molecular weight therapeutic molecules should prove to be useful for fascilitating the penetration of therapeutic molecules to therapeutic targets in the inner ear.

Apoptosis is programmed cell death that involves the controlled dismantling of intracellular components while avoiding inflammation and damage to surrounding cells.^23^ This process is implicated in several inner ear disorders, including drug-induced, noise-induced, age-related, and genetic hearing loss conditions.^24^ Caspases, a family of cysteine proteases, are essential effector molecules for initiating apoptosis.^25–28^ “X-linked inhibitor of apoptosis protein” (XIAP; its molecular weight is 84,000), the most potent member of the inhibitors of apoptosis (IAP) family, interacts physically with caspase-9 at its BIR3 domain and with caspase-3 and caspase-7 at their BIR2 domains, thereby interfering with the apoptosis signaling pathways.^29–31^ XIAP also enhances the survival signal directly by forming a complex with TAB1/TAK1 via its BIR1 domain.^31^ XIAP has a protective effect against several types of inner ear damage. Tabuchi *et al*.^32^ reported on the protective effect of XIAP against gentamicin-induced hair cell damage in an *in vitro* study utilizing rat cochleae, while Wang *et al*.^33,34^ reported that XIAP overexpressed in mice exerted a protective effect against noise-induced and age-related hearing loss in cochlear hair cells and spiral ganglion cells (SGCs). Thus, XIAP appears to have protective effects against drug- and noise-induced hearing loss. Only one report though has examined the treatment efficacy of XIAP *in vivo*: Cooper *et al*.^35^ demonstrated the successful protective treatment effect of XIAP against cisplatin-mediated ototoxicity using an adeno-associated viral vector that was injected into the scala tympani of the cochlea in rats. When considering the possible clinical applications of adeno-associated viral vector-based XIAP treatment for humans, the direct injection of viral vectors into the scala tympani, which is part of the perilymphatic space, may cause inner ear damage; moreover, the direct injection of therapeutic molecules into either the perilymphatic or the endolymphatic spaces of the cochleae have not been performed clinically because of this damage risk. Thus, the need exists for a simpler and safer strategy to administer XIAP into the cochleae. CPP-based XIAP treatment may be such a treatment modality. Additionally, this CPP-based XIAP strategy may be an important treatment modality for administering other therapeutic molecules for treating various inner ear diseases.

Herein, we first examined the safety and efficacy of protein transduction using a CPP consisting of nine arginines (9R-CPP) via the round window (RW) niche. Second, to prove the feasibility of 9R-CPP-based treatment via the RW, we performed 9R-CPP-based XIAP treatment against noise-induced hearing loss in guinea pigs *in vivo*.

## Results

### Protein transduction *in vitro*

HEK293 cells were cultured in medium containing EGFP-9R or EGFP for 24 hours. In the cells, which were cultured with EGFP-9R, EGFP-9R protein was transduced into all of the cytoplasm and into some of the nuclei. In the cells, which were cultured with enhanced green fluorescent protein (EGFP) without 9R, EGFP was not transduced into any cells. Western blotting analyses revealed that the relative GFP intensity in the cells which were treated with EGFP-9R was higher than that in EGFP-treated cells (Supplementary Figure S1a,b). The EGFP protein expression levels at 24 hours after EGFP-9R treatment were equivalent to EGFP protein expression levels at 24 hours after plasmid transfection (Supplementary Figure S1a,b). A chronological study after EGFP-9R treatments revealed that EGFP protein expression levels after EGFP-9R treatments were highest at 12 hours and they still maintain some level of EGFP expression even at 72 hours after the EGFP-9R treatments (Supplementary Figure S1c,d). Additionally, the higher concentrations (10 µmol/l) of EGFP-9R induced longer protein expression periods when compared with lower concentrations (1 µmol/l) (Supplementary Figure S2e). The EGFP protein expression level within the cells after EGFP-9R treatments was approximately similar in level to the EGFP protein level of 2% of the original (100%) EGFP-9R solution (Supplementary Figure S2a). It appears that 2% of the total amount of EGFP moved into the cells via EGFP-9R treatment. WST assay revealed that the viability of both HEK293 cells and MEFs were unaffected even at high dosages (Supplementary Figure S2c,d).

### Protein transduction into the guinea pig cochleae *in vivo*

We performed a single-protein transduction study using the EGFP (s-EGFP Group) and the EGFP-9R (s-EGFP-9R Group) (Figure 1b–d) and a double-protein transduction study using the double EGFP-9R (d-EGFP-9R Group) (Figure 1e). For quantification of EGFP protein transfection, we utilized two strategies: western blot analysis and modified labeling index (mLI) analysis.

**Figure 2:**
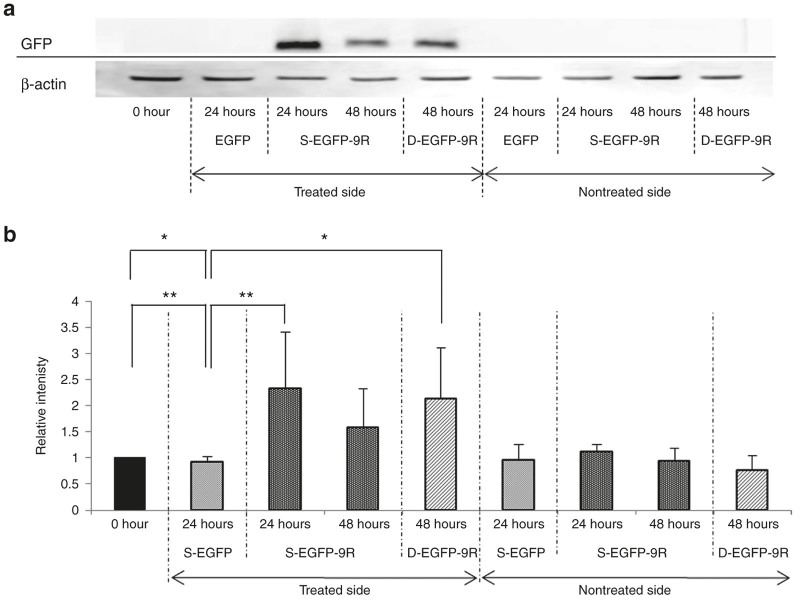
Quantification of protein transduction levels via western blot. (**a**) GFP and β-actin protein expression was detected utilizing western blotting in the s-EGFP-9R and d-EGFP-9R groups. (**b**) Quantification of GFP protein transduction levels was performed utilizing the relative intensity. The relative intensity in s-EGFP-9R Group at 24 hours was significantly higher than that at 0 hours and at 24 hours in the s- enhanced green fluorescent protein (EGFP) groups. The EGFP transduction level at 48 hours in the d-EGFP-9R Group was significantly higher compared with that at 0 hours and that at 24 hours in the s-EGFP Group, while EGFP transduction level at 48 hours in the s-EGFP-9R Group indicates no significant change compared with that at 0 hours and that at 24 hours in the s-EGFP group.

Western blot analyses were performed utilizing whole cochleae. The GFP transduction levels at 24 hours in the s-EGFP Group did not show any significant changes compared with those of the normal non-treated cochleae (referred to as 0 hours in Figure 2a,b). The GFP transduction levels in the s-EGFP-9R Group at 24 hours was significantly higher compared to those at 0 hours and also to those at 24 hours in the s-EGFP Group, while the GFP transduction levels in the s-EGFP-9R Group at 48 hours showed no significant differences compared to those at 0 hours and those at 24 hours in the s-EGFP Group. The GFP transduction levels at 48 hours in the d-EGFP-9R Group was significantly higher compared with those at 0 hours and those at 24 hours in the s-EGFP Group, while GFP transduction levels at 48 hours in the s-EGFP-9R Group indicated no significant changes compared with those at 0 hours and those at 24 hours in the s-EGFP Group (Figure 2). When we compared GFP transduction levels of the s-EGFP-9R and the d-EGFP-9R groups, at the same elapsed time points, after their final treatments; relative intensity at 24 hours in the s-EGFP-9R Group versus that at 48 hours in the d-EGFP-9R Group, no significant differences were detected. These results strongly suggest that a single EGFP-9R application at the RW niche induces significant protein transduction in the cochleae, and that the protein transduction is maintained for at least 24 hours after the placement. A double EGFP-9R application is effective at maintaining protein transduction levels for at least 48 hours.

**Figure 3:**
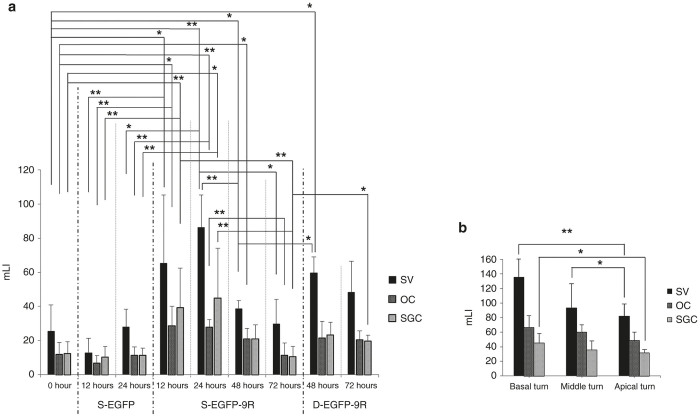
Quantification of protein transduction levels via mLI. (**a**) The mLIs in the s-EGFP, s-EGFP-9R and d-EGFP-9R groups are shown. The mLIs of the treated-side cochleae in the s-EGFP-9R Group peaked at 24 hours, decreased over time, and became approximately equal to that in 0 hours by 72 hours. (**b**) The mLIs at each turn at 24 hours in the s-EGFP-9R Group are shown. The mLI in the SV and SGC at the lower turns was generally higher, versus at the higher turns. Each *n* = 5. *P < 0.05; **P < 0.01.

To assess the localization of the transduced proteins in the cochleae, we performed mLI assessments. The mLIs of the treated cochleae in the s-EGFP-9R Group were significantly higher than those in the s-EGFP Group, in the SVs, OCs (inner hair cells (IHCs), outer hair cells (OHCs), and supporting cells; Supplementary Figure S4a,b), and SGCs at 12 and 24 hours (Figure 3a and Supplementary Figure S4b), while there were no significant differences between the mLIs in the s-EGFP Group and in the normal nontreated cochleae (referred to as 0 hours in Figure 3a). There were no significant differences between the mLI values of the treated-side cochleae at 72 hours in the s-EGFP-9R Group and the mLI at 0 hours (Figure 3a). These findings suggest that the mLIs of the treated cochleae in the s-EGFP-9R Group returned to the baseline level by 72 hours. Additionally, when we compared the mLI at each time point in the s-EGFP-9R Group, the values at 48 hours were significantly lower than those in the SVs at 24 hours, and the mLIs at 72 hours were significantly lower than those in the SGCs at 12 hours and those in the SVs, OCs, and SGCs at 24 hours (Figure 3a).

We assessed the mLI at each turn of the cochlea after 24 hours in the s-EGFP-9R Group. The mLIs of the SVs and SGCs at the basal turns in the s-EGFP-9R Group were significantly higher than those at the apical turns, and the mLIs of the SVs at the middle turns were significantly higher than those at the apical turns (Figure 3b). Thus, in the s-EGFP-9R Group, the mLIs at the basal turns, where the inner surface of the RWM directly faces the scala tympani and which contains the perilymph, was the highest among the three turns.

Regarding the double-protein transduction study, the mLIs of the treated cochleae in the SVs at 48 hours in the d-EGFP-9R Group were significantly higher than those in the SVs at 48 hours in the s-EGFP-9R Group or those at 0 hours (Figure 3a). The mLIs of the treated cochleae in the SGCs at 72 hours in the d-EGFP-9R Group were also significantly higher than those at 72 hours in the SGCs of the s-EGFP-9R Group (Figure 3a). Next, we compared the mLIs in the s-EGFP-9R Group and in the d-EGFP-9R Group at the same elapsed time points after their final treatments. Consequently, the mLIs at 48 and 72 hours in the d-EGFP-9R Group were not significantly different from those at 24 and 48 hours in the s-EGFP-9R Group, respectively. These findings suggest that a double EGFP-9R application is effective at reproducing protein transduction levels of the first application.

### Thickness of the RWM

**Figure 4:**
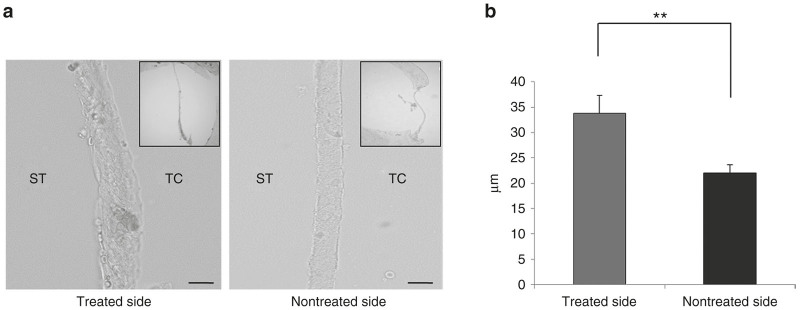
RWM thickness. (**a**) Typical sectional images of the round window membrane (RWM) at 24 hours in the s-EGFP-9R group. The RWM thickness in treated-side cochleae appears to be thicker than that in nontreated cochleae. Scale bars indicate 20 μm. ST: scala tympani; TC: tympanic cavity. (**b**) The thickness of the RWM in the s-EGFP-9R group. The thickness of the RWM in treated-side cochleae was significantly greater than that in nontreated cochleae in the s-EGFP-9R group. Each *n* = 5. **P < 0.01.

At 24 hours in the s-EGFP-9R Group, the thicknesses of the RWMs of the treated-side cochleae were significantly greater than those of the nontreated side cochleae (Figure 4). The increase in RWM thickness may have affected the slight decrease in protein transduction after the second transduction.

### Impact of protein transduction on inner ear function and morphology

To assess the impact of protein transduction on inner ear function, we assessed auditory thresholds, the number of HCs, and the number of SGCs at 28 days in the s-EGFP and s-EGFP-9R Groups. ABR testing revealed that there was no significant change in the auditory thresholds at 4, 12, or 20 kHz between the s-EGFP and s-EGFP-9R Groups (Supplementary Figure S5). Both groups had a normal surface morphology in the OCs (Supplementary Figure S6a). There were no statistically significant differences in the hair cells and SGCs numbers between the groups (Supplementary Figure S6b and S7).

### Protein transduction of XIAP or XIAP-9R *in vivo*

**Figure 5:**
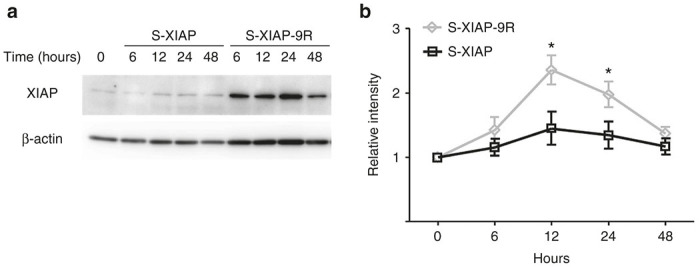
Chronologicanl changes of XIAP protein expression levels. (**a**) Representative western blot results showing the transduction levels of X-linked inhibitor of apoptosis protein (XIAP) in the whole cochleae. (**b**) Quantification of the transduction levels of XIAP in the cochleae. The transduction levels for XIAP in s-XIAP-9R-treated cochleae were significantly higher than those in XIAP-treated cochleae at 12 and 24 hours. Each *n* = 4. *P < 0.05.

Western blot analyses were performed periodically after protein application (Figure 5a). The quantification indicated that the transduction level in the s-XIAP-9R Group increased, with a peak at 12 hours after application (Figure 5b). The transduction level of XIAP in the s-XIAP Group did not show any significant changes.

### Protective effect of XIAP-9R treatment against noise-induced hearing loss

**Figure 6:**
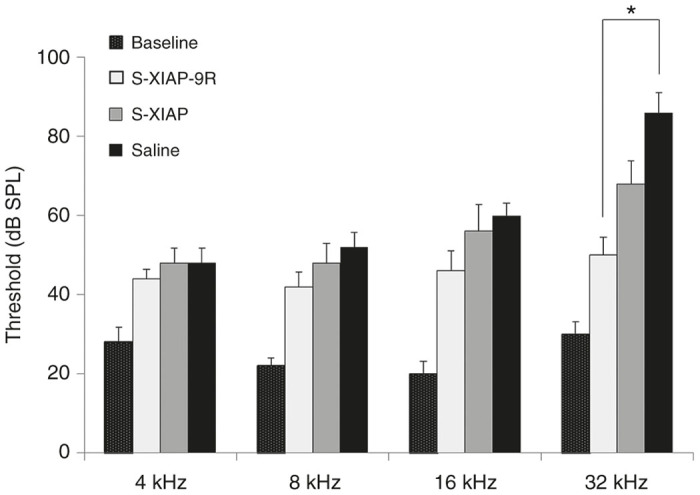
ABR testing results at 14 days after noise exposure. A significant difference was found between the s-XIAP-9R and saline groups at 32 kHz. *P < 0.05.

To examine the feasibility of 9R-CPP-based treatment via the RW niche, we performed XIAP-9R treatment to protect against noise-induced hearing loss. The auditory thresholds in the s-XIAP-9R Group at 14 days after noise exposure were significantly better than those in the Saline Group at 32 kHz, whereas no significant differences were found between the s-XIAP and saline groups (Figure 6).

### Effects of XIAP-9R treatment on hair cell loss

**Figure 7:**
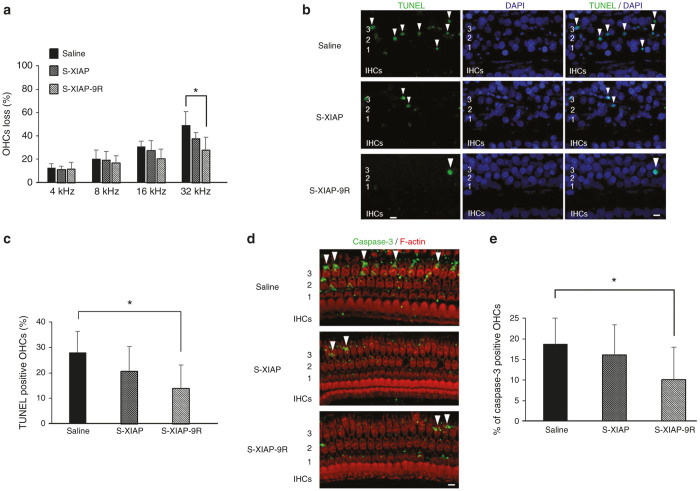
XIAP experiment results. (**a**) Quantification of outer hair cells (OHC) loss at each frequency region at 14 days after treatment is shown. OHC loss at the 32-kHz region in the S-XIAP-9R group was significantly lower than that in the saline group. Each *n* = 5. *P < 0.05. (**b**) Surface preparations from the basal turns of cochleae at 2 days after noise exposure. TUNEL-positive nuclei are indicated by white arrowheads. IHCs: inner hair cells. 1, 2, and 3: first, second, and third rows of OHCs. The scale bar indicates 10 μm. **(c)** Quantification of TUNEL-positive cells in the 32-kHz region. The number of TUNEL-positive cells in the s-XIAP-9R group was significantly lower than that in the saline group. Each *n* = 5. *P < 0.05. (**d**) Surface preparations of the 32-kHz region of a cochlea at 1 day after noise exposure. Cleaved caspase-3-positive cells are indicated by white arrowheads. 1, 2, and 3: first, second, and third rows of OHCs. The scale bar indicates 10 μm. (**e**) Quantification of cleaved caspase-3-positive OHCs from the basal regions. The number of cleaved caspase-3-positive OHCs in the s-XIAP-9R group was significantly lower than that in the saline group. Each *n* = 5. *P < 0.05.

To clarify the morphological effects of the s-XIAP-9R Group, we counted the number of OHCs at 14 days after noise exposure. A surface morphological study revealed that the OHCs loss rate at the base of the cochlea, which corresponds to 32 kHz in the s-XIAP-9R Group, decreased significantly, compared to that in the saline Group, while there was no significant difference in the OHCs loss rate between the s-XIAP and saline Groups (Figure 7a).

### Effects of XIAP-9R treatment on noise-induced apoptosis

A TdT-mediated dUTP nick end labeling (TUNEL) assay was used to assess the mechanism of cochlear HC loss and the protective effect of XIAP-9R against acoustic trauma. Previous studies have shown that TUNEL-positive nuclei were observable as early as 1 hour after noise exposure and that they were observed continuously up to 72 hours after noise exposure.^36^ Thus, we performed TUNEL labeling for HCs and supporting cells of the OC in the basal turns of the cochleae in each group at 48 hours after noise exposure (Figure 7b). TUNEL-positive nuclei were clearly apparent in the region of the OHCs in the saline Group. The TUNEL-positive nuclei showed the characteristic changes of apoptosis, including a shrunken appearance and the formation of micronuclei. There were fewer TUNEL-positive nuclei in the s-XIAP and s-XIAP-9R Groups (Figure 7b). Subsequently, a quantitative analysis revealed that the number of TUNEL-positive OHC nuclei decreased significantly in the s-XIAP-9R Group, but not in the s-XIAP Group, compared with that in the saline Group (Figure 7c). These results suggest that XIAP-9R treatment against noise-induced cochlear damage had a protective effect via the inhibition of apoptosis.

To further examine the mechanism of the antiapoptotic effects of XIAP in noise exposure in the cochleae, we examined cleaved caspase-3 in the cochleae at 24 hours after noise exposure. It has been reported that cleaved caspase-3 is detectable in the cochleae from immediately after noise exposure up to 2 days after noise exposure,^37,38^ and it is therefore a prime target for the antiapoptotic effects of XIAP. Cleaved caspase-3-positive OHCs were found more frequently in the basal parts of the OCs than in the middle and apical turns; this is consistent with the finding that apoptotic OHCs were found most frequently at the basal turns after noise exposure (data not shown). Cleaved caspase-3-positive cells were found less frequently in the s-XIAP-9R Group than in the s-XIAP Group or in the saline Groups. The number of cleaved caspase-3-positive OHCs in the s-XIAP-9R Group was significantly lower than in the saline group (Figure 7d,e). These results suggest that an apoptotic mechanism, via caspase-3, was suppressed by the XIAP-9R treatment.

## Discussion

Western blot analyses revealed that a single EGFP-9R application at the RW niche induced significant protein transduction in the cochleae. The protein transduction in the whole cochleae was maintained for at least 24 hours after the application when compared with the nontreated cochleae, whereas, a single EGFP application did not induce significant protein transduction. In the XIAP-9R study, the western blot analysis data similarly revealed that XIAP transduction was maintained for at least 24 hours after the treatment. Double EGFP-9R applications were effective at maintaining protein transduction levels for at least 48 hours after the first applications. To assess the localization of the transduced proteins in the cochleae after 9R-CPP-mediated protein transfer via the RWM, we utilized mLI assessment, which is a “photoshop-based image analysis method,^39^ which has been utilized for assessing protein expression levels in *in vivo* and in *in vitro* experiments.^40–48^ Consequently, we found that protein transduction signals were significantly detectable in all assessed locations (the SV, SGC, and OC regions) until 24 hours after the EGFP-9R application, when compared with nontreated cochleae. While at 48 hours after the EGFP-9R applications, significant protein transduction levels were detectable in the SV and SGC regions in the cochleae. In the double application study, significant protein transduction signals were detectable in the SVs at 48 hours after the initial applications, when compared with the nontreated cochleae. Thus, sequential changes of the protein transduction levels found in our mLI analysis in the EGFP-9R experiment were similar to those found in the western blot analysis; in both analyses, we could detect significant protein transduction signals in the cochleae for at least 24 hours after the single EGFP-9R treatments, and for at least 48 hours after the double EGFP-9R treatments, but according to the results, there were some minor differences in the sequential changes of the protein transduction levels between the western blot analysis data and the mLI analysis data, (for example, at 48 hours after the single EGFP-9R application, significant protein transduction signals were not detectable in western blot analysis, but significant signals were detectable in two of the three locations in the cochleae with mLI analysis), we think that these differences reflect differences in the target samples: whole cochleae in the western blot analyses and selected locations in the cochlea in the mLI analyses. We therefore maintain that protein transduction via the RWM utilizing 9R-CPP is a proven, valid, and reliable protein transduction strategy into the cochleae.

Our *in vitro* studies revealed that the EGFP protein expression levels at 24 hours after EGFP-9R treatment were equivalent to the EGFP protein expression levels at 24 hours after plasmid transfection via cationic lipid-mediated transfection. Recently, Zuris *et al*.^49^ have reported successful protein transfection into the cochlear tissues via cationic lipid mediated transfection. Although they injected target proteins and cationic lipid into the scala media, the direct injection into the cochlea has a potential risk to cause inner ear damage. Additionally, Zuris *et al*.^49^ have found cytotoxic effects in both in vitro and in vivo studies when utilizing the higher concentration of cationic lipid. Contrarily, 9R-CPP-based protein transfection did not show any cytotoxic effects in either HEK293 cells or MEFs even under at higher concentrations of 9R-CPP. Additionally, in *in vivo* studies, 9R-CPP-based protein transfection did not show any cytotoxic effects at either the highest concentration levels of EGFP-9R or XIAP-9R, which were available to us. Thus, when considering clinical applications of protein transduction into the cochleae, 9R-CPP-based protein transduction appears to be superior to cationic lipid-mediated transfection because of the lack of cytotoxicity with 9R-CPP-based transfections.

There are two known routes for the diffusion of substances from the tympanic cavity, the normal air space surrounding the bony cochleae, to the perilymphatic space in the cochleae of rodents. One is via the RWM and the other is via the bony wall of the cochlea. It has been reported that gentamicin and steroid hormones placed on the RW niche of guinea pigs are distributed into the perilymphatic space of the cochlea. The concentration at the basal turn of the cochlea was 1,000-fold higher than at the apical turn.^50^ This suggests that substances placed on the RW niche pass through the RWM and diffuse into the perilymphatic space from the basal turn to the apical turn. In guinea pigs, the lateral bony wall of the cochlear apex is thinner than at the basal turn.^51^ Additionally, a lacuno-canalicular system has been described in the cochlear walls of both humans and mice.^52^ These anatomical structures in guinea pigs appear to induce a higher concentration of a substance at the apical turn when the tympanic cavity is filled with the substance.^53^ The existence of the two routes (via the RWM and via the bony wall of the cochlea) may have contributed to the distribution of the substances used in the present study. The EGFP intensities at the basal turns in the cochleae were significantly higher than at the apical turns after placement of an EGFP-9R-soaked gelatin sponge. Considering our distribution pattern of EGFP intensity, we concluded that EGFP-9R probably diffuses mainly via the RWM and becomes distributed in the perilymphatic space in the cochlea, rather than via the cochlear bony wall.

Although the general mechanism by which CPPs enter cells has yet to be determined, it has been suggested that they use direct penetration and endocytosis for cellular internalization.^54,55^ Additionally, CPPs appear to use paracellular pathways to pass through the mucosa and brain endothelial cells.^54,56^ In the present study, EGFP-9R and XIAP-9R, which were placed at the RW niche, probably reached the perilymphatic space using these cellular and paracellular pathways. Generally, it is known that substances which exist in the perilymphatic space in the scala tympani reach the modious, the Rosenthal canals and the OC by passing successively through the canaliculi perforantes.^57^ It is also known that there is communication between the scala tympani and the scala vestibuli via the spiral ligament and the stria vascularis.^58–60^ In our experiments, EGFP-9R and XIAP-9R, which were placed at the RW niche, may have extended to each part in the cochlea via these routes.

To assess the feasibility of 9R-CPP-based treatment via the RW niche, we performed a protection study using XIAP-9R against noise-induced hearing loss in guinea pigs *in vivo*. XIAP-9R applied topically to the RWM successfully prevented auditory deterioration caused by noise-induced cochlear damage at higher frequencies. The presumed mechanism underlying this protective effect is that 9R-CPP coupled to XIAP facilitated XIAP permeability across the RWM and diffusion into the cochlear tissues, including OHCs; subsequently, 9R-XIAP achieved a protective effect against noise-induced hearing loss by suppressing the caspase-3 pathway. Considering this, 9R-XIAP treatment via the RW niche could be a simple and promising treatment modality for decreasing cochlear damage in sensorineural hearing loss caused by an apoptotic mechanism. The finding that XIAP-9R showed significant protective effects at higher frequencies but a limited protective effect at lower frequencies may limit the clinical application of XIAP-9R treatment. This finding may have been caused by differences in the XIAP distribution pattern dependent upon each specific cochlear turn; in the s-EGFP-9R Group, the EGFP intensity at lower turns showed significantly higher EGFP transduction. The concentration of XIAP-9R was 0.1 mg/ml, while the concentration of EGFP-9R was 4 mg/ml. Application of such a lower concentration of XIAP-9R at the RW niche should have lowered the local concentration of XIAP in the cochlea, especially at the higher turns. Tünnemann *et al*.^55^ has also revealed that the transduction ability of arginines can be affected not only by their concentration, but also by the number of consecutive residues. Considering these points, optimization of the concentration levels and the number of consecutive arginine residues may increase the efficacy of transduction, and this in turn should increase the therapeutic efficacy of cargos coupled with the CPP. Furthermore, in the noise-induced hearing loss model used in the present study, the protective effect was lower at lower frequencies than at higher frequencies. Our model produced auditory deterioration primarily at higher frequencies and less so at lower frequencies. This difference in the auditory deterioration pattern may explain our finding of a reduced protective effect at lower frequencies in XIAP-9R-treated guinea pigs. To further explore this issue, we need to optimize the conditions for CPPs and to evaluate the effects of CPP treatment on other inner ear damage models, including models that produce auditory deterioration at lower frequencies.

A single 9R-CPP-based protein transduction via the RW niche exerts more efficient protein transduction compared with protein transduction without 9R-CPP for at least 24 hours after treatment. When we look at time points of the peaks of protein expression levels in our studies, there were some differences: 24 hours in *in vivo* mLI study and 12 hours in WB results of *in vivo* study and in XIAP study. We think that this difference probably cones from difference of assessing methods (mLI versus WB) and location of the samples (second turns of the cochleae versus whole cochleae). In either case, 12 to 24 hours of expression periods after a single 9R-CPP-based protein transduction may limit the usefulness of this method. There are two possible solutions for this issue. Our *in vitro* study revealed that a higher concentration level of 9R-CPP induced longer protein expression periods. In our *in vivo* study, we utilized the highest concentration of 9R-CPPs which we had available in our institution, though, if we might have used a higher concentration of 9R-CPPs, probably we could have achieved longer expression periods. Additionally, repeated applications may also help to extend expression periods. In our study, following an additional EGFP-9R application at 24 hours after the first, EGFP protein transduction levels at 48 hours after the first application were significantly higher compared to those in the controls. Additionally, regarding the EGFP protein transduction levels at the same elapsed time after one versus two applications, no significant differences were observed between the s-EGFP and s-EGFP-9R groups. These findings indicate that multiple consecutive applications through the RW niche are effective in terms of extending the expression duration. However, when we compared EGFP protein transduction levels at the same elapsed time between single EGFP-9R application and double EGFP-9R application in detail, the EGFP protein transduction level at 48 hours in the d-EGFP-9R group was somewhat lower than that at 24 hours in the s-EGFP-9R group. This slight decrease in EGFP protein transduction level may be caused by thickening of the RWM: the thickness of the RWM at 24 hours in the treated cochleae was significantly greater than that in the nontreated cochleae. The thickening of the RWM might be a limiting factor in transduction efficacy in more multiple consecutive applications. This thickening phenomenon might have been caused by the gelatin sponge,^61^ which was used as a carrier of the EGFP-9R. If so, the use of other materials instead of gelatin sponges, or the application of 9R-CPP-conjugated cargos without other materials, may diminish the thickening of the RWM. Consequently, these strategies may increase the transduction efficacy by repeated applications.

Many studies have shown that CPPs are efficient carriers of bioactive cargos, including proteins, peptides, and oligonucleotides. Although the most common application of CPPs is the transduction of proteins and peptides, other applications include CPP-mediated oligonucleotide delivery.^62,63^ The non-specific nature of CPP-mediated delivery may be a drawback when considering clinical applications. Laakkonen *et al*.^64^ reported that LyP-1 peptide, a CPP, induced strong accumulation in primary MDAMB-435 breast cancer xenografts after intravenous peptide injections. Tan *et al*.^65^ reported that a CPP conjugated with an anti-HER-2/neu peptide mimetic (AHNP), an ErbB2 extracellular domain-binding peptide, preferentially targeted ErbB2-overexpressing in breast cancer cells. The Matsushita^20^ and Kemp *et al*.^66^ reported that the addition of a nuclear localization signal (NLS) resulted in delivery of the peptide exclusively to the nuclear compartment of the cells. Thus, the attachment of such homing peptides or other targeting motifs to CPPs may increase retention in specific tissues, cell types, or intracellular locations.

We utilized one octave band noise centered at 4 kHz at 116 dB sound pressure level (SPL) for 2 hours to generate a noise-induced hearing loss model. This noise exposure level induced deterioration of the auditory thresholds, mainly at high frequencies. It has been well known that there are differences in the frequencies of noise and the location of subsequent cochlear damage. Hair cell loss at the basal turns seem to be independent of the noise frequency band used in exposure.^67^ The upward spread of cochlear damage with respect to the exposure spectrum is typical of an acoustic injury.^68^ It has also been well known that the hearing impairment level depends on the sound intensity, the types of sound, and the susceptibility of the animals. Thus, the relationship between the noise frequency and the location of the subsequent damage is reasonable and logical.

When considering clinical applications of CPP mediated treatments, in humans, it is relatively easy to access the RW niche via the tympanic membrane, and repeated applications via the tympanic membrane are also feasible. Although we used gelatin sponges as a carrier for EGFP-9R in the current study, we suggest that the repeated application of 9R-CPP-conjugated cargos via eardrops without using a carrier through an incised tympanic membrane could be a clinically applicable method.

In summary, we demonstrated that the use of a 9R-CPP via the RW niche is an effective and safe method for protein transduction into guinea pig cochleae. We also demonstrated some effectiveness of repeated applications. Additionally, we showed that 9R-CPP-based treatment is feasible, using XIAP-9R in guinea pigs. Thus, we believe that 9R-CPP-based transduction via the RW niche is a promising potential method for the delivery of therapeutically relevant molecules into the cochlea in humans.

## Materials and Methods

### Ethics statement

All animal experiments were approved by the Committee on the Use and Care of Animals at Kumamoto University and the National Defense Medical College. They were performed according to accepted veterinary standards.

### Recombinant proteins

EGFP and EGFP fused to 9R (EGFP-9R) were prepared as described previously.^20,69^ Briefly, the constructed plasmids were transfected into BL21-DE3 *Escherichia coli* cells, and then protein expression was induced by 0.2 mmol/l isopropyl 1-thio-β-D-galactopyranoside. The proteins were purified using TALON resin (Clontech, Mountain View, CA) and stored at −80 °C after dialysis against phosphate-buffered saline (PBS). For the purification of recombinant XIAP and XIAP fused to 9R (XIAP-9R), mouse full-length Xiap cDNA was amplified utilizing polymerase chain reaction using appropriate linker primers and subcloned into the EcoRI-XhoI sites of pET21a(+) (Novagen, Madison, WI) or the BamHI-EcoRI sites of pET21a(+), fused with the 9R sequence at the carboxyl terminus. Similarly, the proteins were generated and stored.

### Experimental protocol *in vivo*

Regarding the EGFP and EGFP-9R experiments, we used Hartley guinea pigs, weighing 250–300 g each (Kyudo, Saga, Japan). The EGFP and EGFP-9R experiments involved three groups: single EGFP application (s-EGFP group); single EGFP-9R application (s-EGFP-9R group); and, double EGFP-9R applications (d-EGFP-9R group), in which the second application was performed 24 hours after the first. EGFP and EGFP-9R applications were achieved in the following manner. Guinea pigs were anesthetized via the intraperitoneal administration of 10 mg/kg of xylazine (Bayer; Shawnee Mission, KS) and 40 mg/kg of ketamine (Daiichisankyo, Tokyo, Japan) in 0.9% NaCl. The animals underwent a postauricular incision, and then the mastoid bullae were opened. Subsequently, a gelatin sponge of 5–10 mm^3^ (Astellas Pharma, Tokyo, Japan) that was soaked in 5 μl of EGFP (24.4 mg/ml) or EGFP-9R (4.0 mg/ml) was placed on the RW niche of the right ear, followed by the intraperitoneal administration of 20 mg/kg of chloramphenicol (Daiichisankyo, Tokyo, Japan). The holes in the mastoid bullae were sealed immediately with muscle tissue and the skin was closed. In each group, the cochleae were extracted after the treatment at each time point: the animals were euthanized with deep anesthesia using an overdose of xylazine and ketamine. Normal nontreated cochleae were used as controls and the data of them were expressed as the data at 0 hours. At 28 days after treatment, auditory functional analyses were performed in the s-EGFP Group and in the s-EGFP-9R Group. Subsequently, the animals in those groups were euthanized for further morphological analyses.

The XIAP and XIAP-9R experiments involved three groups of 4-week-old Hartley guinea pigs (Japan SLC, Shizuoka, Japan): a single XIAP application (s-XIAP group); a single XIAP-9R application (s-XIAP-9R Group); and a saline application (saline group). Under general anesthesia with ketamine and xylazine, a gelatin sponge of 5–10 mm^3^ soaked in XIAP or XIAP-9R at a concentration of 0.1 mg/ml was placed on the RW niche in the right ear of the animals at 12 hours before noise exposure. As a control, a gelatin sponge immersed in saline was used. Subsequently, auditory functional analyses and morphological analyses were then performed.

### Western blotting

In the EGFP and EGFP-9R experiments, five cochlear samples were evaluated at each time point. Proteins were extracted with an extraction kit (Thermo Fisher Scientific, Waltham, MA). The supernatants of the homogenates from each cochlea were subjected to sodium dodecyl sulfate-polyacrylamidegel electrophoresis (SDS-PAGE), and the proteins in the gel were transferred to Polyvinylidene Difluoride (PVDF) membranes (Bio-rad, Hercules, CA). The blots were immunoreacted with Horse radish peroxidase-conjugated anti-GFP (Bio-rad) and with Horse radish peroxidase-conjugated anti-β-actin (Bio-rad) antibodies, and the protein bands were then visualized using a specific chemiluminescence detection system (Bio-rad). The immunoblot signals were detected using an LAS4000 digital imaging system (Fujifilm, Tokyo, Japan). The detected bands were analyzed with Image J software (NIH, Bethesda, MD). For quantification of protein transduction levels, we utilized the relative intensity obtained by dividing GFP intensity by the β-actin intensity; subsequently, the values were normalized to that at 0 hours.

In the XIAP and XIAP-9R experiments, four cochlear samples were evaluated at each time point. The supernatants of the homogenates from each cochlea were subjected to SDS-PAGE. The proteins were transferred to an Immobilon-P membrane (Millipore, Billerica, MA). The blots were immunoreacted with anti-XIAP (BD Biosciences, San Jose, CA) and with anti-β-actin (Sigma, St. Louis, MO) antibodies, and the protein bands were then visualized using a specific chemiluminescence detection system (Pierce, Rockford, IL). The immunoblot signals were detected using an LAS4000 digital imaging system (Fujifilm). The detected protein bands were then analyzed using Image Quant TL software (GE Healthcare, Little Chalfont, UK). The GFP intensity was divided by the β-actin intensity; subsequently, the values were normalized to control cochlear samples taken from nontreated animals.

### mLI

In the s-EGFP and s-EGFP-9R Groups, whole cochleae were harvested and fixed in 4% PFA (Wako, Osaka, Japan) for 12 hours at 4 °C. The inner ears were decalcified in disodium ethylenediaminetetraacetic acid (EDTA) for 14 days. The cochleae were embedded in optimal cutting temperature (OCT) medium (Sakura Finetek Japan, Tokyo, Japan) and sectioned serially at a thickness of 8 μm utilizing cryostat (GMI, Bellevue, WA). The cryosectioned slices were fixed with 4% PFA and blocked with 0.1% Triton-X (IBI Scientific Kapp Court Peosta, IA) in PBS, and then incubated with Hoechst 33258 Dye (Molecular Probes) for 30 seconds for nuclear staining. During each process, the tissues were washed three times for 5 minutes each with PBS. The samples were examined under a BZ-9000 fluorescence microscope (Keyence, Osaka, Japan), and the images were captured and stored on a computer. We used the mLI to evaluate the EGFP protein transduction levels in each cryosectioned slice of the cochleae. The original labeling index method was reported by Lehr.^39^ The mLI method used is described as follows: each image was captured at a resolution of 1,360 × 1,024 pixels at the same photographic exposure condition; tissue-free areas outside of the bony cochleae were then selected using the Magic Wand tool in Adobe Photoshop (Adobe Systems, San Jose, CA); and, the tolerance level was then adjusted to be able to select the entire outside areas of the bony cochleae. Thus, the determined tissue-free areas were used as the background. Subsequently, each stained area in the organ of Corti (OC), SGCs, and stria vascularis (SV) was determined similarly. These images were converted to 8-bit grayscale images. The optical densities of the background areas and each stained area were assessed using the histogram tool in Adobe Photoshop; consequently, the mean staining intensity and the number of pixels in each area were determined. The final staining intensity was calculated as the difference between the mean staining intensity and the mean background intensity. Next, the staining ratios were calculated as the ratios of the number of pixels in each stained area to that in the entire image. These measuring processes of mLI were performed in a blinded manner.

### Noise exposure

Guinea pigs were anesthetized by an intraperitoneal injection of ketamine (50 mg/kg) and medetomidine (1.0 mg/kg). They were exposed to one octave band noise centered at 4 kHz at 116 dB sound pressure level (SPL) for 2 hours in a ventilated sound exposure chamber. The sound chamber was fitted with speakers (Model 2380A; JBL, Northridge, CA) driven by a noise generator (DANAC-31; Dana Japan, Tokyo, Japan) and a power amplifier (D-45; Crown International, Elkhart, IN). Sound levels were calibrated (Type 6224 precision sound level meter; Aco Instruments, Tokyo, Japan) at multiple locations within the sound chamber to ensure uniformity of the stimulus.

### Auditory thresholds

Auditory thresholds were measured using the ABR (System 3; Tucker-Davis Technologies, Alachua, FL). The animals were anesthetized by the intraperitoneal administration of xylazine and ketamine. Electrodes were placed beneath the pinna of the treated ear and at the vertex just below the surface of the skin, and then the ground electrode was placed under the contralateral ear. An average of 1,024 sweeps was calculated for 4, 8, and 12 kHz in the s-EGFP and s-EGFP-9R Groups, and for 4, 8, 16, and 32 kHz in the s-XIAP and s-XIAP-9R Groups. The stimulus levels near the threshold were varied in 5-dB steps, and the threshold was defined as the lowest level at which waves in the ABR could be clearly detected by visual inspection. In the s-EGFP and s-EGFP-9R Groups, the hearing thresholds were measured at 28 days after protein transduction. In the s-XIAP and s-XIAP-9R Groups, the ABR was measured 1 day before noise exposure and on 14 days after noise exposure.

### Morphological analyses

In the s-EGFP and s-EGFP-9R groups, the thicknesses of the RWM at 24 hours after treatment were assessed. The bilateral cochleae were serially cryosectioned to a thickness of 8 μm in each group. Nomarski images of the whole RWM were captured using a BZ-9000 microscope (Keyence, Osaka, Japan) and then they were stored on a computer at a density of 96 dpi. The center part of the RWM was cut out from the captured RWM images. Subsequently, the lengths and areas of the RWM in the cut-off images were measured utilizing ImageJ software (NIH, Bethesda, MD). The values obtained by dividing the areas by the length were utilized for statistical analyses.

For hair cell counting, in the s-EGFP and s-EGFP-9R Groups, the cochleae were removed from the temporal bonesat 28 days after treatment. After fixation overnight at 4 °C, the bony capsules and lateral walls of the cochleae were removed. The OCs were incubated with Texas Red-X phalloidin (Molecular Probes) for 30 minutes after blocking with 0.3% Triton-X in PBS for 10 minutes. During each process, the tissues were washed three times for 5 minutes each with PBS. The surface images of the OCs were captured and stored on a computer. The numbers of IHCs and OHCs in 300 μm lengths of the OCs in each turn were counted, and the survival rate of the hair cells (HCs) was measured. In the s-XIAP, s-XIAP-9R, and saline groups, similarly, OCs were harvested on 14 days after noise exposure. After immersion in the fixative agent overnight at 4 °C, the temporal bones were decalcified in 0.1 M EDTA (pH 7.4) containing 5% sucrose and stirred at 4 °C for 2 days. After decalcification, the cochlea was microdissected. The OCs were incubated with primary antibodies overnight at 4 °C after blocking with 0.1% Triton-X in PBS supplemented with 5% goat serum for 1 hour. During each process, the tissues were washed three times for 20 minutes each with PBS. Primary antibodies were detected using secondary antibodies and viewed under confocal fluorescence microscopy (Nikon C1 system; Tokyo, Japan). Hair cells were identified using anti-myosin VIIa antibodies (Proteus Biosciences, San Diego, CA) as the primary antibody and Alexa 350 (Invitrogen, Carlsbad, CA) as the secondary antibody. ImageJ software (NIH) was used to measure the total length of the cochlear whole mounts, and a cochlear frequency map was computed to precisely localize IHCs based on frequency-specific regions. ImageJ plugin (http://www.masseyeandear.org/research/otolaryngology/investigators/laboratories/eaton-peabody-laboratories/epl-histology-resources/imagej-plugin-for-cochlear-frequency-mapping-in-whole-mounts) that translates cochlear position into specific frequency according to the published map of the guinea pig.^70^ High-power images of frequency-specific regions according to the computed frequency map were assembled and analyzed. The numbers of IHCs and OHCs in 100 μm were counted in four segments in the cochlea.

In the same manner as described for the hair cell counts, the whole-mounted samples were stained to detect DNA fragmentation in the nuclei of apoptotic cells using the TUNEL technique with an ApopTag Plus Fluorescein In Situ Apoptosis Detection Kit (Millipore) according to the manufacturer’s recommended protocol. Briefly, residues of digoxigenin-deoxyribonucleotide triphosphate were added to the 3’-OH ends of the DNA using terminal deoxynucleotidyl transferase. The incorporated digoxigenin-nucleotides were then immunostained with a FITC-conjugated anti-digoxigenin antibody from the TUNEL kit. The tissues were counterstained with DAPI. The FITC and DAPI fluorescent signals were observed by confocal fluorescence microscopy using the Nikon C1 system. To quantify TUNEL-positive OHCs, the total number of OHCs and the number of TUNEL-positive OHCs were counted in each image. Counts were obtained from five cochleae. For each condition, the OHC counts were obtained from three locations in the basal turns of each cochlea.

After whole-mount preparation of the cochleae, caspase-3-positive cells were identified using cleaved caspase-3 antibodies (Cell Signaling Technology, Danvers, MA) as the primary antibody and Alexa 488 (Invitrogen) as the secondary antibody. Subsequently, counterstaining was performed using F-actin (Invitrogen). The numbers of caspase-3-positive OHCs per 100 μm in the basal turns were counted in s-XIAP, the s-XIAP-9R, and in the saline groups.

### Statistical analyses

The data were analyzed statistically using the Mann-Whitney *U*-test. Data are presented in the text and figures as means ± standard error. The statistical significance level was set at *P* < 0.05.

## Notes

The first three authors contributed equally to this work.

## References

[bib1] Liu, X, Zheng, G, Wu, Y, Shen, X, Jing, J, Yu, T et al. (2013). Lead exposure results in hearing loss and disruption of the cochlear blood-labyrinth barrier and the protective role of iron supplement. Neurotoxicology 39: 173–181. 24144481 10.1016/j.neuro.2013.10.002

[bib2] Liu, H, Hao, J and Li, KS (2013). Current strategies for drug delivery to the inner ear. Acta Pharm Sin B 3: 86–96.

[bib3] Swan, EE, Mescher, MJ, Sewell, WF, Tao, SL and Borenstein, JT (2008). Inner ear drug delivery for auditory applications. Adv Drug Deliv Rev 60: 1583–1599. 18848590 10.1016/j.addr.2008.08.001 PMC2657604

[bib4] McCall, AA, Swan, EE, Borenstein, JT, Sewell, WF, Kujawa, SG and McKenna, MJ (2010). Drug delivery for treatment of inner ear disease: current state of knowledge. Ear Hear 31: 156–165. 19952751 10.1097/AUD.0b013e3181c351f2 PMC2836414

[bib5] Bowe, SN and Jacob, A (2010). Round window perfusion dynamics: implications for intracochlear therapy. Curr Opin Otolaryngol Head Neck Surg 18: 377–385. 20808222 10.1097/MOO.0b013e32833d30f0

[bib6] Goycoolea, MV and Lundman, L (1997). Round window membrane. Structure function and permeability: a review. Microsc Res Tech 36: 201–211. 9080410 10.1002/(SICI)1097-0029(19970201)36:3<201::AID-JEMT8>3.0.CO;2-R

[bib7] Borkholder, DA, Zhu, X and Frisina, RD (2014). Round window membrane intracochlear drug delivery enhanced by induced advection. J Control Release 174: 171–176. 24291333 10.1016/j.jconrel.2013.11.021 PMC3925065

[bib8] Goycoolea, MV, Muchow, D and Schachern, P (1988). Experimental studies on round window structure: function and permeability. Laryngoscope 98(6 Pt 2 Suppl 44): 1–20. 10.1288/00005537-198806001-00002 3287079

[bib9] Goycoolea, MV, Muchow, DD, Sirvio, LM, Winandy, RM, Canafax, DM and Hueb, M (1992). Extended middle ear drug delivery. Acta Otolaryngol Suppl 493: 119–126. 1636411

[bib10] Lundman, L, Bagger-Sjöbäck, D, Juhn, SK and Morizono, T (1992). Pseudomonas aeruginosa exotoxin A and Haemophilus influenzae type b endotoxin. Effect on the inner ear and passage through the round window membrane of the chinchilla. Acta Otolaryngol Suppl 493: 69–76. 1636425

[bib11] Green, M and Loewenstein, PM (1988). Autonomous functional domains of chemically synthesized human immunodeficiency virus tat trans-activator protein. Cell 55: 1179–1188. 2849509 10.1016/0092-8674(88)90262-0

[bib12] Frankel, AD and Pabo, CO (1988). Cellular uptake of the tat protein from human immunodeficiency virus. Cell 55: 1189–1193. 2849510 10.1016/0092-8674(88)90263-2

[bib13] Vocero-Akbani, A, Lissy, NA and Dowdy, SF (2000). Transduction of full-length Tat fusion proteins directly into mammalian cells: analysis of T cell receptor activation-induced cell death. Methods Enzymol 322: 508–521. 10914043 10.1016/s0076-6879(00)22046-6

[bib14] Schmidt, N, Mishra, A, Lai, GH and Wong, GC (2010). Arginine-rich cell-penetrating peptides. FEBS Lett 584: 1806–1813. 19925791 10.1016/j.febslet.2009.11.046

[bib15] Schwarze, SR, Ho, A, Vocero-Akbani, A and Dowdy, SF (1999). *In vivo* protein transduction: delivery of a biologically active protein into the mouse. Science 285: 1569–1572. 10477521 10.1126/science.285.5433.1569

[bib16] Eguchi, A, Meade, BR, Chang, YC, Fredrickson, CT, Willert, K, Puri, N et al. (2009). Efficient siRNA delivery into primary cells by a peptide transduction domain-dsRNA binding domain fusion protein. Nat Biotechnol 27: 567–571. 19448630 10.1038/nbt.1541 PMC2694965

[bib17] Takenobu, T, Tomizawa, K, Matsushita, M, Li, ST, Moriwaki, A, Lu, YF et al. (2002). Development of p53 protein transduction therapy using membrane-permeable peptides and the application to oral cancer cells. Mol Cancer Ther 1: 1043–1049. 12481427

[bib18] Futaki, S (2005). Membrane-permeable arginine-rich peptides and the translocation mechanisms. Adv Drug Deliv Rev 57: 547–558. 15722163 10.1016/j.addr.2004.10.009

[bib19] Futaki, S (2002). Arginine-rich peptides: potential for intracellular delivery of macromolecules and the mystery of the translocation mechanisms. Int J Pharm 245: 1–7. 12270237 10.1016/s0378-5173(02)00337-x

[bib20] Matsushita, M, Tomizawa, K, Moriwaki, A, Li, ST, Terada, H and Matsui, H (2001). A high-efficiency protein transduction system demonstrating the role of PKA in long-lasting long-term potentiation. J Neurosci 21: 6000–6007. 11487623 10.1523/JNEUROSCI.21-16-06000.2001 PMC6763134

[bib21] Wender, PA, Mitchell, DJ, Pattabiraman, K, Pelkey, ET, Steinman, L and Rothbard, JB (2000). The design, synthesis, and evaluation of molecules that enable or enhance cellular uptake: peptoid molecular transporters. Proc Natl Acad Sci USA 97: 13003–13008. 11087855 10.1073/pnas.97.24.13003 PMC27168

[bib22] Lindsay, MA (2002). Peptide-mediated cell delivery: application in protein target validation. Curr Opin Pharmacol 2: 587–594. 12324264 10.1016/s1471-4892(02)00199-6

[bib23] McIlwain, DR, Berger, T and Mak, TW (2013). Caspase functions in cell death and disease. Cold Spring Harb Perspect Biol 5: a008656. 23545416 10.1101/cshperspect.a008656 PMC3683896

[bib24] Op de Beeck, K, Schacht, J and Van Camp, G (2011). Apoptosis in acquired and genetic hearing impairment: The programmed death of the hair cell. Hear Res 281: 18–27. 21782914 10.1016/j.heares.2011.07.002 PMC3341727

[bib25] Nicholson, DW, Ali, A, Thornberry, NA, Vaillancourt, JP, Ding, CK, Gallant, M et al. (1995). Identification and inhibition of the ICE/CED-3 protease necessary for mammalian apoptosis. Nature 376: 37–43. 7596430 10.1038/376037a0

[bib26] Salvesen, GS. (2012) Caspases: cell signaling by proteolysis. In: Handbook of Cell Signaling. 2nd edn. Bradshaw, RA and Dennis, EA. Oxford, pp 1297–1302.

[bib27] Thornberry, NA and Lazebnik, Y (1998). Caspases: enemies within. Science 281: 1312–1316. 9721091 10.1126/science.281.5381.1312

[bib28] Wang, J and Lenardo, MJ (1997). Molecules involved in cell death and peripheral tolerance. Curr Opin Immunol 9: 818–825. 9492984 10.1016/s0952-7915(97)80184-7

[bib29] Bratton, SB, Lewis, J, Butterworth, M, Duckett, CS and Cohen, GM (2002). XIAP inhibition of caspase-3 preserves its association with the Apaf-1 apoptosome and prevents CD95- and Bax-induced apoptosis. Cell Death Differ 9: 881–892. 12181739 10.1038/sj.cdd.4401069

[bib30] Suzuki, Y, Nakabayashi, Y, Nakata, K, Reed, JC and Takahashi, R (2001). X-linked inhibitor of apoptosis protein (XIAP) inhibits caspase-3 and -7 in distinct modes. J Biol Chem 276: 27058–27063. 11359776 10.1074/jbc.M102415200

[bib31] Obexer, P and Ausserlechner, MJ (2014). X-linked inhibitor of apoptosis protein - a critical death resistance regulator and therapeutic target for personalized cancer therapy. Front Oncol 4: 197. 25120954 10.3389/fonc.2014.00197 PMC4112792

[bib32] Tabuchi, K, Pak, K, Chavez, E and Ryan, AF (2007). Role of inhibitor of apoptosis protein in gentamicin-induced cochlear hair cell damage. Neuroscience 149: 213–222. 17869439 10.1016/j.neuroscience.2007.06.061

[bib33] Wang, J, Tymczyszyn, N, Yu, Z, Yin, S, Bance, M and Robertson, GS (2011). Overexpression of X-linked inhibitor of apoptosis protein protects against noise-induced hearing loss in mice. Gene Ther 18: 560–568. 21228883 10.1038/gt.2010.172

[bib34] Wang, J, Menchenton, T, Yin, S, Yu, Z, Bance, M, Morris, DP et al. (2010). Over-expression of X-linked inhibitor of apoptosis protein slows presbycusis in C57BL/6J mice. Neurobiol Aging 31: 1238–1249. 18755525 10.1016/j.neurobiolaging.2008.07.016

[bib35] Cooper, LB, Chan, DK, Roediger, FC, Shaffer, BR, Fraser, JF, Musatov, S et al. (2006). AAV-mediated delivery of the caspase inhibitor XIAP protects against cisplatin ototoxicity. Otol Neurotol 27: 484–490. 16791039 10.1097/01.mao.0000202647.19355.6a

[bib36] Wang, J, Ruel, J, Ladrech, S, Bonny, C, van de Water, TR and Puel, JL (2007). Inhibition of the c-Jun N-terminal kinase-mediated mitochondrial cell death pathway restores auditory function in sound-exposed animals. Mol Pharmacol 71: 654–666. 17132689 10.1124/mol.106.028936

[bib37] Hu, BH, Henderson, D and Nicotera, TM (2002). F-actin cleavage in apoptotic outer hair cells in chinchilla cochleas exposed to intense noise. Hear Res 172: 1–9. 12361861 10.1016/s0378-5955(01)00361-6

[bib38] Nicotera, TM, Hu, BH and Henderson, D (2003). The caspase pathway in noise-induced apoptosis of the chinchilla cochlea. J Assoc Res Otolaryngol 4: 466–477. 14534835 10.1007/s10162-002-3038-2 PMC3202741

[bib39] Lehr, HA, Mankoff, DA, Corwin, D, Santeusanio, G and Gown, AM (1997). Application of photoshop-based image analysis to quantification of hormone receptor expression in breast cancer. J Histochem Cytochem 45: 1559–1565. 9358857 10.1177/002215549704501112

[bib40] Hosseini, A, Baker, JL, Tokin, CA, Qin, Z, Hall, DJ, Stupak, DG et al. (2014). Fluorescent-tilmanocept for tumor margin analysis in the mouse model. J Surg Res 190: 528–534. 24923630 10.1016/j.jss.2014.05.012 PMC4201840

[bib41] Reifenberg, K, Cheng, F, Twardowski, L, Küpper, I, Wiese, E, Bollmann, F et al. (2013). T cell-specific overexpression of TGFß1 fails to influence atherosclerosis in ApoE-deficient mice. PLoS One 8: e81444. 24339930 10.1371/journal.pone.0081444 PMC3855303

[bib42] Wang, H, Li, Y, Yu, W, Ma, L, Ji, X and Xiao, W (2015). Expression of the receptor for advanced glycation end-products and frequency of polymorphism in lung cancer. Oncol Lett 10: 51–60. 26170976 10.3892/ol.2015.3200 PMC4487081

[bib43] Chen, YJ, Lam, J, Gregory, CR, Schrepfer, S and Wulff, H (2013). The Ca²⁺-activated K⁺ channel KCa3.1 as a potential new target for the prevention of allograft vasculopathy. PLoS One 8: e81006. 24312257 10.1371/journal.pone.0081006 PMC3843675

[bib44] Lau, DH, Shipp, NJ, Kelly, DJ, Thanigaimani, S, Neo, M, Kuklik, P et al. (2013). Atrial arrhythmia in ageing spontaneously hypertensive rats: unraveling the substrate in hypertension and ageing. PLoS One 8: e72416. 24013508 10.1371/journal.pone.0072416 PMC3754972

[bib45] Eckle, T, Hughes, K, Ehrentraut, H, Brodsky, KS, Rosenberger, P, Choi, DS et al. (2013). Crosstalk between the equilibrative nucleoside transporter ENT2 and alveolar Adora2b adenosine receptors dampens acute lung injury. FASEB J 27: 3078–3089. 23603835 10.1096/fj.13-228551 PMC3714574

[bib46] Jin, J, Zhang, X, Lu, Z, Li, Y, Lopes-Virella, MF, Yu, H et al. (2014). Simvastatin inhibits lipopolysaccharide-induced osteoclastogenesis and reduces alveolar bone loss in experimental periodontal disease. J Periodontal Res 49: 518–526. 24117880 10.1111/jre.12132 PMC3979522

[bib47] Padler-Karavani, V, Hurtado-Ziola, N, Chang, YC, Sonnenburg, JL, Ronaghy, A, Yu, H et al. (2014). Rapid evolution of binding specificities and expression patterns of inhibitory CD33-related Siglecs in primates. FASEB J 28: 1280–1293. 24308974 10.1096/fj.13-241497 PMC3929681

[bib48] Doan, PL, Himburg, HA, Helms, K, Russell, JL, Fixsen, E, Quarmyne, M et al. (2013). Epidermal growth factor regulates hematopoietic regeneration after radiation injury. Nat Med 19: 295–304. 23377280 10.1038/nm.3070 PMC3594347

[bib49] Zuris, JA, Thompson, DB, Shu, Y, Guilinger, JP, Bessen, JL, Hu, JH et al. (2015). Cationic lipid-mediated delivery of proteins enables efficient protein-based genome editing *in vitro* and in vivo. Nat Biotechnol 33: 73–80. 25357182 10.1038/nbt.3081 PMC4289409

[bib50] Tinling, SP and Chole, RA (1994). Apical cochlear nerve exposed to perilymph in the gerbil and rat. Hear Res 73: 203–208. 8188549 10.1016/0378-5955(94)90236-4

[bib51] Plontke, SK, Biegner, T, Kammerer, B, Delabar, U and Salt, AN (2008). Dexamethasone concentration gradients along scala tympani after application to the round window membrane. Otol Neurotol 29: 401–406. 18277312 10.1097/MAO.0b013e318161aaae PMC2587453

[bib52] Zehnder, AF, Kristiansen, AG, Adams, JC, Kujawa, SG, Merchant, SN and McKenna, MJ (2006). Osteoprotegrin knockout mice demonstrate abnormal remodeling of the otic capsule and progressive hearing loss. Laryngoscope 116: 201–206. 16467704 10.1097/01.mlg.0000191466.09210.9a PMC2563156

[bib53] Mikulec, AA, Plontke, SK, Hartsock, JJ and Salt, AN (2009). Entry of substances into perilymph through the bone of the otic capsule after intratympanic applications in guinea pigs: implications for local drug delivery in humans. Otol Neurotol 30: 131–138. 19180674 10.1097/mao.0b013e318191bff8 PMC2729139

[bib54] Shi, NQ, Qi, XR, Xiang, B and Zhang, Y (2014). A survey on “Trojan Horse” peptides: opportunities, issues and controlled entry to “Troy”. J Control Release 194: 53–70. 25151981 10.1016/j.jconrel.2014.08.014

[bib55] Tünnemann, G, Ter-Avetisyan, G, Martin, RM, Stöckl, M, Herrmann, A and Cardoso, MC (2008). Live-cell analysis of cell penetration ability and toxicity of oligo-arginines. J Pept Sci 14: 469–476. 18069724 10.1002/psc.968

[bib56] Ohtake, K, Maeno, T, Ueda, H, Natsume, H and Morimoto, Y (2003). Poly-L-arginine predominantly increases the paracellular permeability of hydrophilic macromolecules across rabbit nasal epithelium *in vitro*. Pharm Res 20: 153–160. 12636152 10.1023/a:1022485816755

[bib57] Salt, AN and Plontke, SK (2009). Principles of local drug delivery to the inner ear. Audiol Neurootol 14: 350–360. 19923805 10.1159/000241892 PMC2820328

[bib58] Salt, AN, Ohyama, K and Thalmann, R (1991). Radial communication between the perilymphatic scalae of the cochlea. II: Estimation by bolus injection of tracer into the sealed cochlea. Hear Res 56: 37–43. 1769923 10.1016/0378-5955(91)90151-x

[bib59] Plontke, SK, Wood, AW and Salt, AN (2002). Analysis of gentamicin kinetics in fluids of the inner ear with round window administration. Otol Neurotol 23: 967–974. 12438864 10.1097/00129492-200211000-00026

[bib60] Saijo, S and Kimura, RS (1984). Distribution of HRP in the inner ear after injection into the middle ear cavity. Acta Otolaryngol 97: 593–610. 6464711 10.3109/00016488409132937

[bib61] Bunzen, DL, Lins, N, Leal, Mde C, Lira, MM and Caldas Neto, Sda S (2014). Middle ear packing materials: comparison between absorbable hemostatic gelatine sponge and sugarcane biopolymer sponge in rats. Braz J Otorhinolaryngol 80: 237–244. 25153109 10.1016/j.bjorl.2013.08.001 PMC9535480

[bib62] Matsushita, M, Noguchi, H, Lu, YF, Tomizawa, K, Michiue, H, Li, ST et al. (2004). Photo-acceleration of protein release from endosome in the protein transduction system. FEBS Lett 572: 221–226. 15304352 10.1016/j.febslet.2004.07.033

[bib63] Michiue, H, Tomizawa, K, Wei, FY, Matsushita, M, Lu, YF, Ichikawa, T et al. (2005). The NH2 terminus of influenza virus hemagglutinin-2 subunit peptides enhances the antitumor potency of polyarginine-mediated p53 protein transduction. J Biol Chem 280: 8285–8289. 15611109 10.1074/jbc.M412430200

[bib64] Laakkonen, P, Akerman, ME, Biliran, H, Yang, M, Ferrer, F, Karpanen, T et al. (2004). Antitumor activity of a homing peptide that targets tumor lymphatics and tumor cells. Proc Natl Acad Sci U S A 101: 9381–9386. 15197262 10.1073/pnas.0403317101 PMC438985

[bib65] Tan, M, Lan, KH, Yao, J, Lu, CH, Sun, M, Neal, CL et al. (2006). Selective inhibition of ErbB2-overexpressing breast cancer *in vivo* by a novel TAT-based ErbB2-targeting signal transducers and activators of transcription 3-blocking peptide. Cancer Res 66: 3764–3772. 16585203 10.1158/0008-5472.CAN-05-2747

[bib66] Kemp, BE, Cheng, HC and Walsh, DA (1988). Peptide inhibitors of cAMP-dependent protein kinase. Methods Enzymol 159: 173–183. 2842583 10.1016/0076-6879(88)59018-3

[bib67] Pouyatos, B, Gearhart, CA, Nelson-Miller, A, Fulton, S and Fechter, LD (2009). Selective vulnerability of the cochlear Basal turn to acrylonitrile and noise. J Toxicol 2009: 908596. 20130768 10.1155/2009/908596 PMC2809326

[bib68] Yamashita, D, Jiang, HY, Schacht, J and Miller, JM (2004). Delayed production of free radicals following noise exposure. Brain Res 1019: 201–209. 15306254 10.1016/j.brainres.2004.05.104

[bib69] Miwa, T, Minoda, R, Kaitsuka, T, Ise, M, Tomizawa, K and Yumoto, E (2011). Protein transduction into the mouse otocyst using arginine-rich cell-penetrating peptides. Neuroreport 22: 994–999. 22045255 10.1097/WNR.0b013e32834da8f8

[bib70] Tsuji, J and Liberman, MC (1997). Intracellular labeling of auditory nerve fibers in guinea pig: central and peripheral projections. J Comp Neurol 381: 188–202. 9130668

