# Supplementary material for: Protein transduction therapy into cochleae via the round window niche in guinea pigs
Source: Mol Ther Methods Clin Dev. 2016 Aug 17;3:16055–. doi: 10.1038/mtm.2016.55 (PMC4988354; doi:10.1038/mtm.2016.55)
Supplement: Supplementary Methods [file mtm201655-s1.doc]

**Protein transduction therapy into cochleae via the round window niche in guinea pigs**

Hiroki Takeda^1^, Takaomi Kurioka^2^, Taku Kaitsuka^3^, Kazuhito Tomizawa^3^, Takeshi Matsunobu^2^,　Farzana Hakim^3^, Kunio Mizutari^2^, Toru Miwa^1^, Takao Yamada^1^, Momoko Ise^1^, Akihiro Shiotani^2^, Eiji Yumoto^1^ and Ryosei Minoda^1^

^1^Department of Otolaryngology-Head and Neck Surgery, Kumamoto University, 1-1-1 Honjo, Kumamoto, Japan

^2^Department of Otolaryngology, National Defense Medical College, 3-2 Namiki, Tokorozawa, Japan

^3^Department of Molecular Physiology, Kumamoto University, 1-1-1 Honjo, Kumamoto, Japan

**Supplementary Methods**

**Protein Transfer Efficiency of 9R-CPP**

HEK293 cells were cultured with EGFP-9R until the evaluation time points. Subsequently, the cells were washed with PBS, scraped, and lysed in SDS sample buffer (63 mM Tris-HCl, pH 6.8, 2% SDS, 5% β-mercaptoethanol, 1.7% glycerol, 0.01% bromophenol blue). After sonication, the lysates were boiled for 4 min. Samples were loaded and subjected to SDS-PAGE, transferred to a nitrocellulose membrane with the iBlot ® gel transfer device (Invitrogen), and then blocked for nonspecific binding in Blocking One (Nacalai Tesque, Kyoto, Japan). Membranes were incubated with either mouse anti-β-actin (1:10,000; Merk Millipore, Billerica, MA) or mouse anti-GFP antibody (1:2,000; Medical & Biological Laboratories, Nagoya, Japan) overnight at 4ºC. Then membranes were washed and incubated for 1 hr with HRP-conjugated anti-rabbit or mouse immunogloblins antibody (1:2,000; Dako). After washing, membranes were incubated with Amersham ECL Prime (GE Healthcare) and the immunoreactive proteins were visualized with ImageQuart400 (GE Healthcare) and quantified with Image J software® (NIH).

Similarly, HEK293 cells were transfected with pEGFP-C1 plasmid (Clontech) using Lipofectamine 2000® reagent (Invitrogen). At 6 hrs after the treatments, the medium was replaced by growth medium and cells were grown for an additional 18 hrs. Then cells were lysed and subjected to western blotting as described above. Empty plasmid was utilized as a control.

To evaluate transfection efficacy into cells via 9R-CPP treatment, we performed WB analyses utilizing serially diluted EGFP-9R solutions without any transfections. Then, the WB results, which were obtained from the serially diluted EGFP-9R solutions without any transfections, were compared with the results which were obtained from the cells which underwent EGFP-9R treatments: the concentration of EGFP-9R which was utilized for cell transfection in vitro, was defined as 100% (original) concentration of EGFP-9R.

To evaluate the relationship between 9R-CPP concentrations and protein expression periods after 9R-CPP treatments, we assessed EGFP expression levels at 6, 48 and 96 hrs after 9R-CPP transfection utilizing 10 µM and 1 µM of EGFP-9R.

**Cytotoxicity Tests of 9R-CPP**

HEK293 cells and mouse embryonic fibroblasts (MEFs) were treated with EGFP-9R protein for 24 hrs and then a WST assay was performed using the Cell counting kit (Dojindo) according to the manufacturer’s instructions. The concentrations of 9R-CPP which we utilized for these in vitro studies were lower than those which we utilized for our in vivo study, because after the placement of the 9R-CPP (4mg/ml) at the round window niche, the local concentrations of 9R-CPP in the intra-cochlear cells should be significantly lower than the original 9R-CPP concentration at the round window niche.

**SGC Counts**

The whole cochleae were obtained from the temporal bones of the s-EGFP and s-EGFP-9R groups at 28 days after treatment. The cochleae were fixed in 4% paraformaldehyde for 12 hrs at 4°C. The inner ears were decalcified in disodium EDTA for 14 days. For cryostat sectioning, the cochleae were embedded in OCT medium (Sakura Finetek Japan, Tokyo, Japan) and sectioned serially at a thickness of 8 μm. The sections were incubated with an anti-β-tubulin (TUJ1) monoclonal antibody (Abcam Inc., Cambridge, UK) after fixing and blocking, and then incubated overnight at 4°C with the primary antibody. Tetramethylrhodamine goat anti-mouse IgG (Thermo Fisher Scientific, Waltham, MA, USA) was applied for 1 hr as the secondary antibody. Subsequently, the slices were incubated with Hoechst 33258 Dye (Molecular Probes) for 30 secs. During each process, the tissues were washed three times for 5 min each with PBS. The images were captured and stored on a computer. Cells double-positive for Hoechst and TUJ1 at each turn were counted as SGCs in three randomly selected sections per animal. The average number of double-positive cells was used for further statistical analyses.

**Supplementary figures and figure legends**


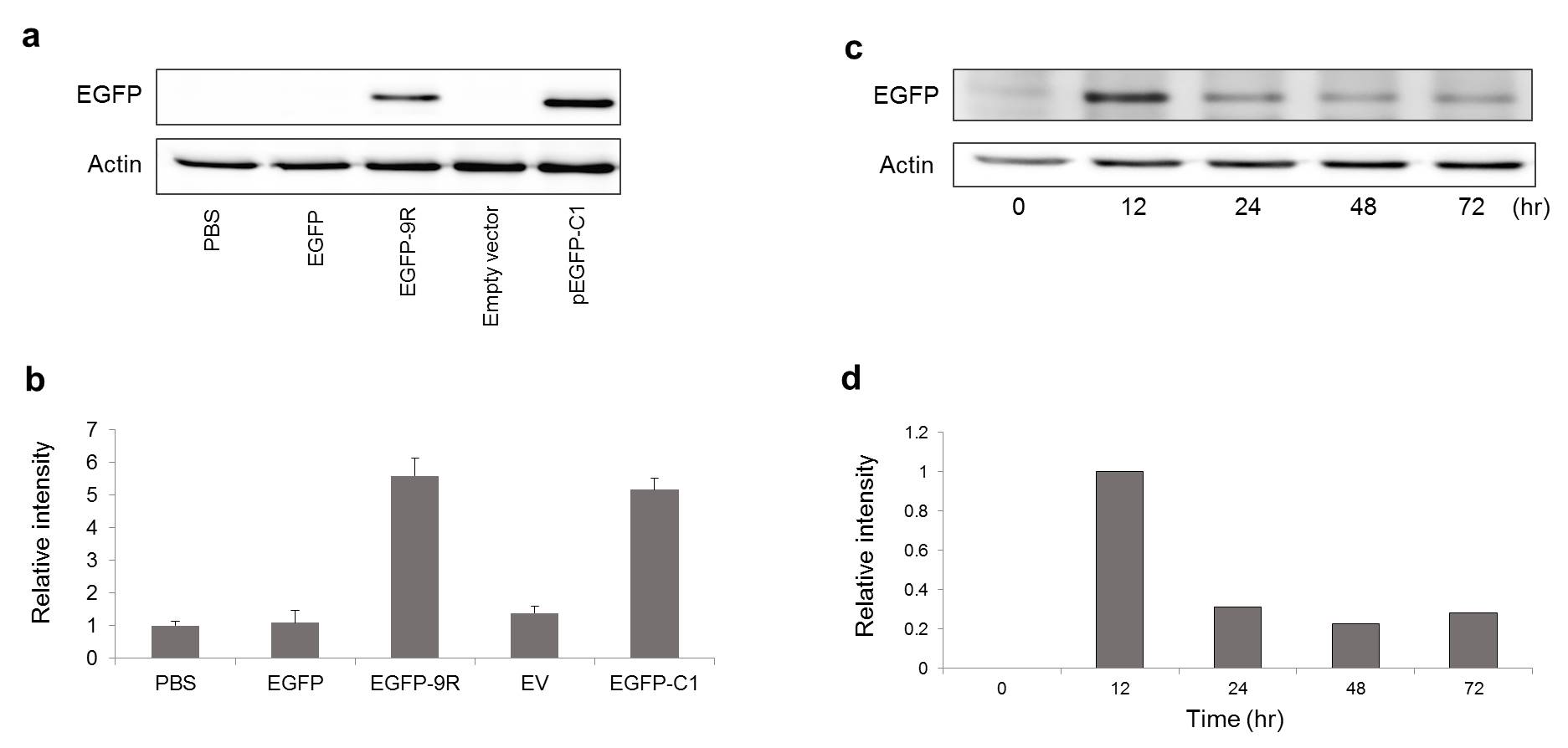


**S1.**

**Protein transfection utilizing 9R-CPP and gene transfection utilizing plasmids vectors in HEK293 cells. (a)** Representative western blot results are shown. Each evaluation was performed at 24 hrs after the treatment. **(b)** The quantification of EGFP protein transduction levels at 24 hrs after each treatment. The relative intensity in EGFP-9R treated cells was equivalent to proteins expressed by plasmid transfections which was achieved via lipofection. Each *n* = 4. **(c)** A chronological change in western blot results after EGFP-9R treatments is shown. **(d)** The relative intensity of EGFP protein expression after EGFP-9R treatments was highest at 12 hrs and it still maintained some level of intensity even at 72 hrs. Each *n* = 1.

**
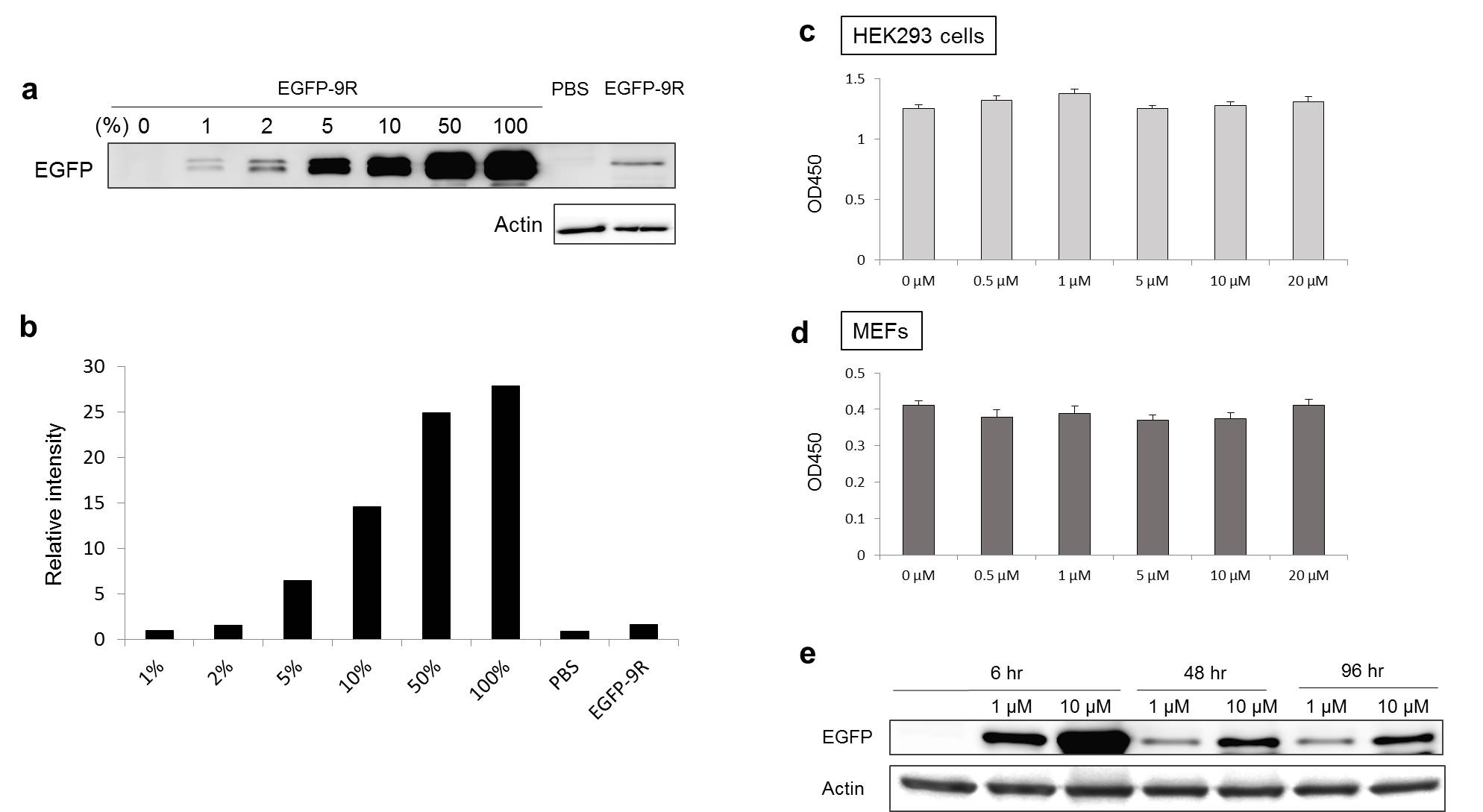
**

**S2.**

(a) Protein transfer efficiency of 9R-CPP in HEK293 cells. This is the western blot results showing EGFP protein expression levels in diluted EGFP-9R solutions and the EGFP protein expression level in HEK293 cells to which EGFP was transfected via EGFP-9R for 24 hrs. The EGFP-9R concentration in the culture medium was defined as 100% concentration of EGFP-9R. (b) Quantification of the (a) data shows that the EGFP protein level within the cells was at an approximately similar level as the EGFP protein level of 2% of the EGFP-9R solution. Each *n* = 1.

(c)(d) Cytotoxicity tests of 9R-CPP. The WST assay results indicate the viability of both HEK293 cells and MEFs were unaffected, even at high dose EGFP-9R treatments. Each *n* = 4.

(e) 9R-CPP concentrations and protein expression periods after 9R-CPP treatments. The western blot results show that the higher concentration (10 µM) of EGFP-9R induced a longer protein expression period when compared with the lower concentration (1 µM).


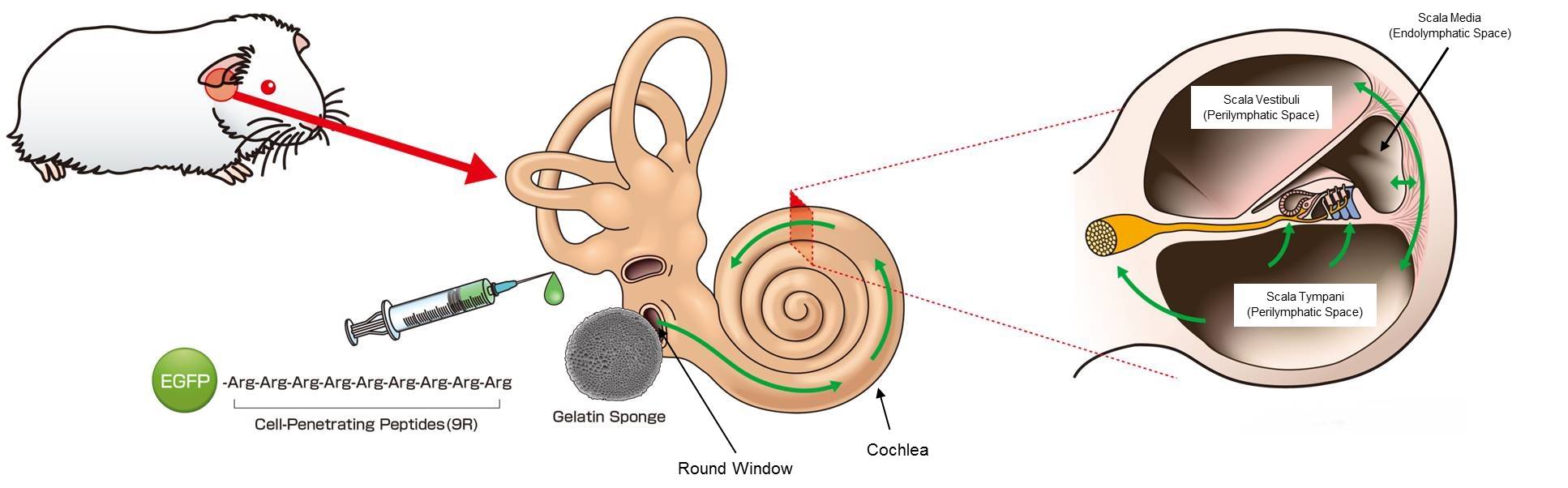


**S3.**

This figure shows an outline of the EGFP experiments. After a postauricular incision, a gelatin sponge that was soaked in EGFP or EGFP-9R was placed on the round window niche of the left ear of the guinea pig. After EGFP-9R application, EGFP was transduced to various cochlear tissues probably through the perilymphatic space. This image was provided by Ray creation co Ltd., Japan under an Open Access License.


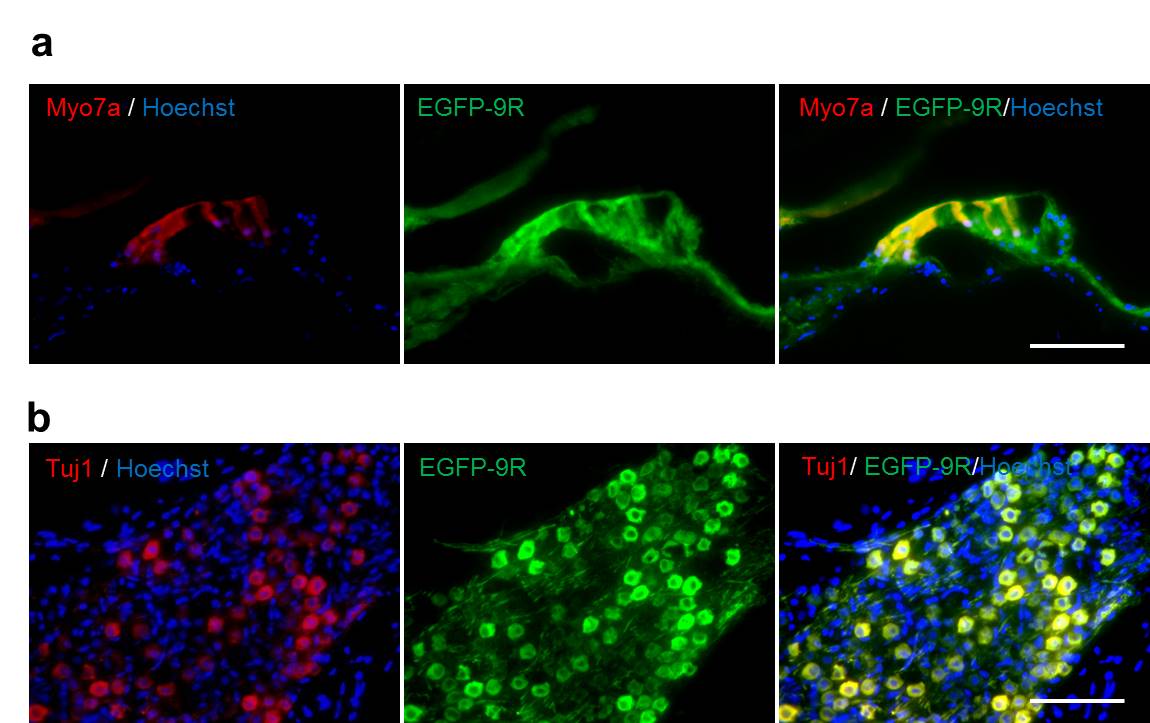


**S4.**

The images of the OCs (a) and the SGC (b) of the middle turns of the cochleae at 24 hrs after EGFP-9R treatment. **(a)** The images were immunostained with Myo7a (red) and Hoechst (blue). EGFP-9R (green) signals are detectable at inner hair cells, at outer hair cells and at supporting cells. **(b)** The images were immunostained with Tuj1 (red) and Hoechst (blue). EGFP-9R positive expression was detectable at Tuj1-positive cells. The scale bars indicate 50 μm.


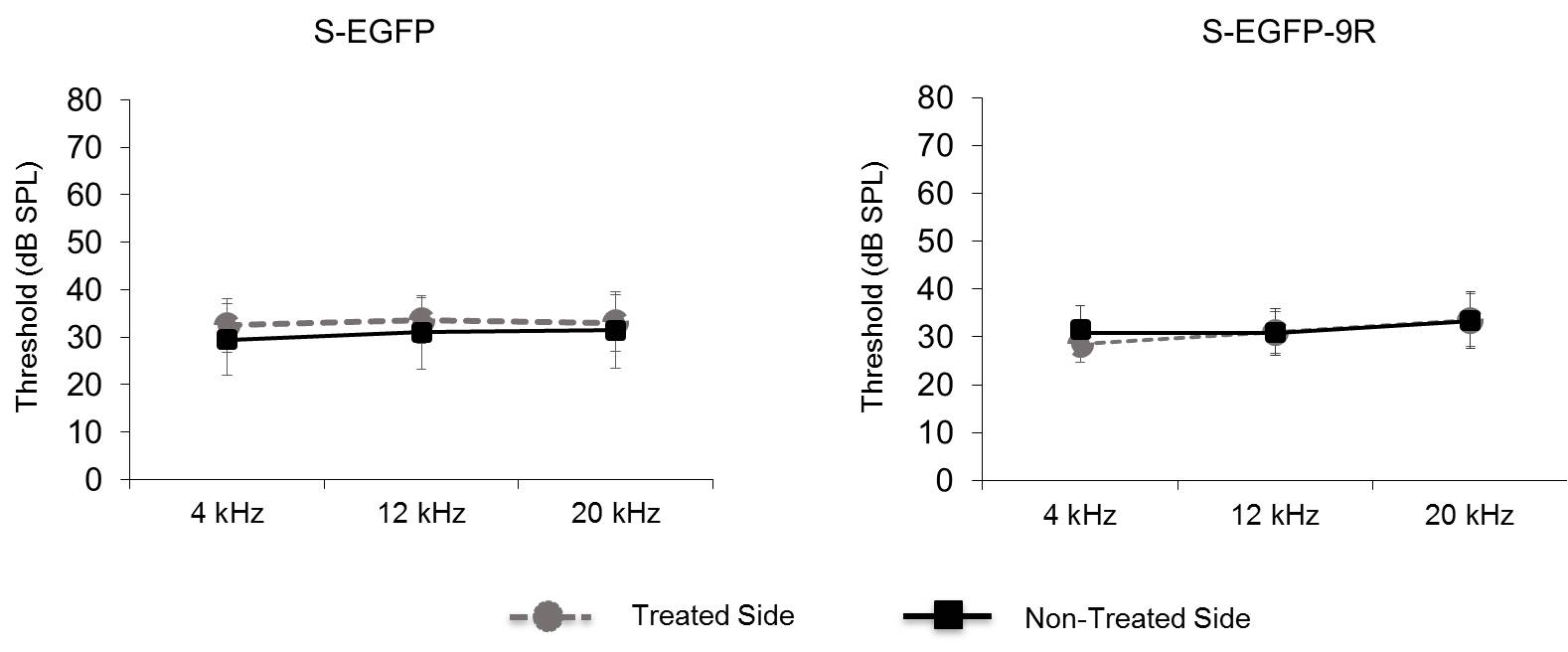


**S5.**

Auditory thresholds at 28 days. No significant differences were noted between the auditory thresholds on the treated and non-treated sides at 28 days in either of the s-EGFP or the s-EGFP-9R groups. Each *n* = 10.


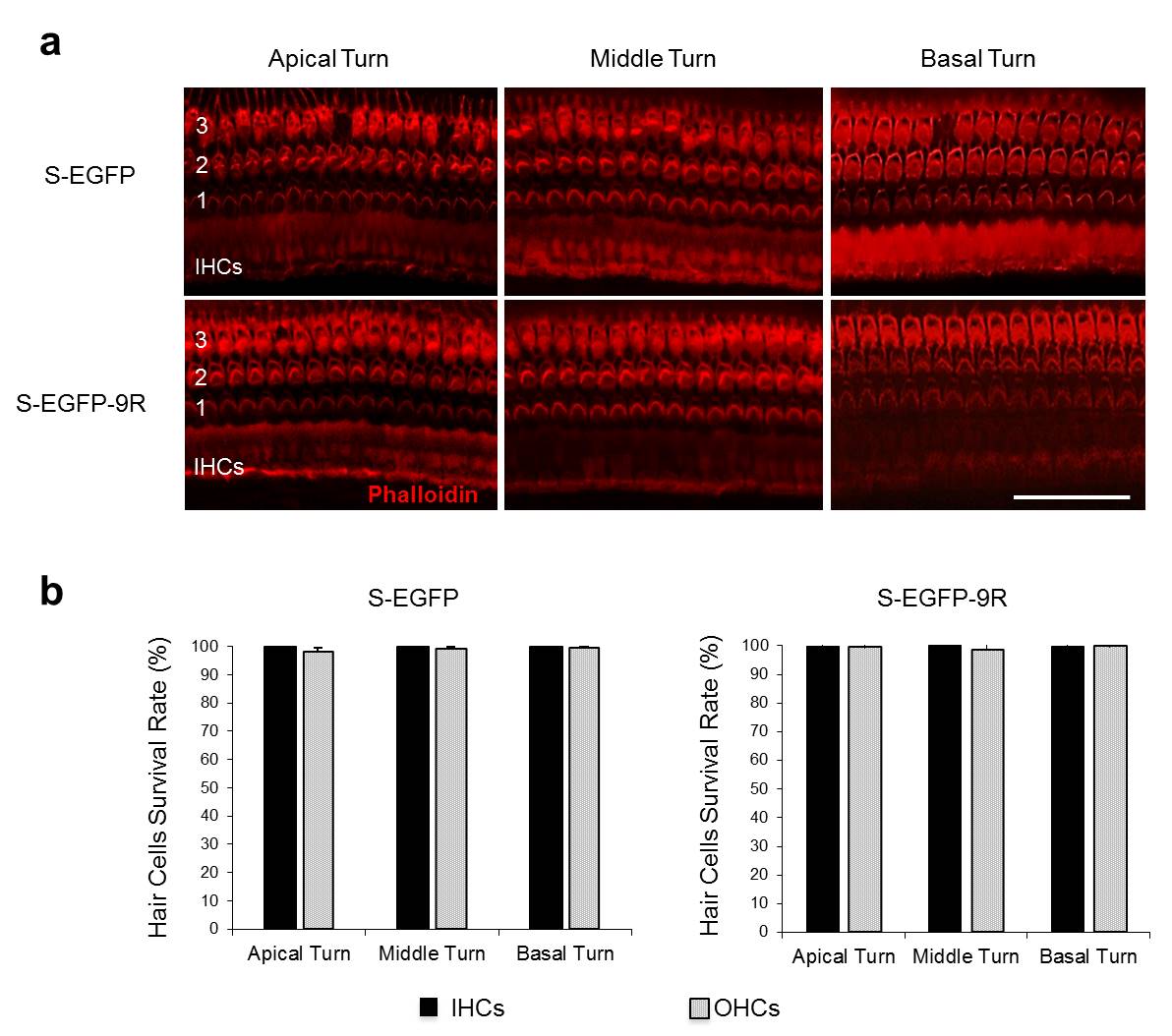


**S6.**

Surface morphology of the OCs at 28 days in the s-EGFP and s-EGFP-9R groups. **(a)** The surface of the OC at 28 days appeared to be almost normal in the s-EGFP and s-EGFP-9R groups. The scale bar indicates 50 μm. **(b)** Survival rates of the cochlear hair cells at 28 days. No significant differences were found between the survival rates of the IHCs and those of the OHCs in the s-EGFP and s-EGFP-9R groups. Each *n* = 5.

**
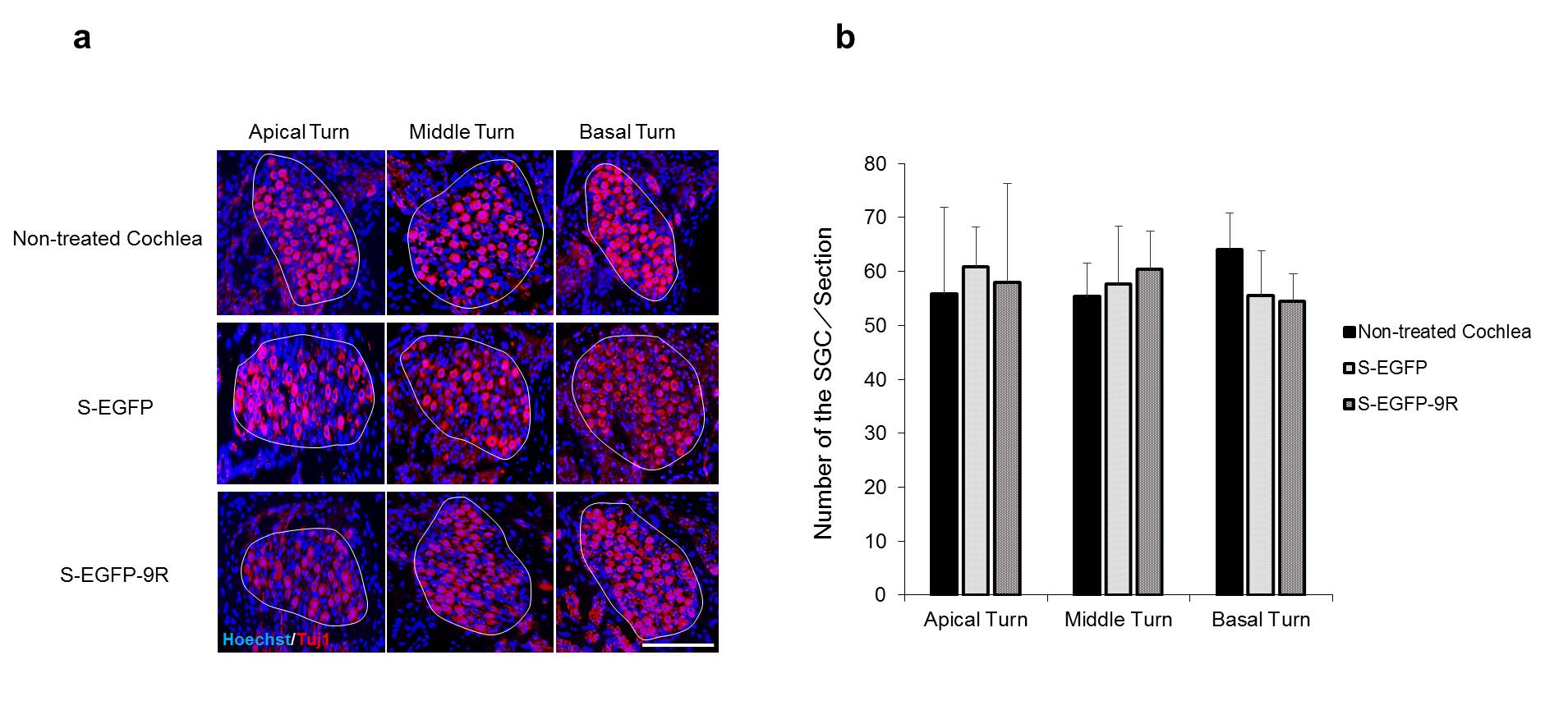
**

**S7.**

**(a)** Representative images of SGC at 28 days. The images were immunostained with TUJ-1 (red color) and Hoechst (blue color). The SGC at 28 days appear to be almost normal in the non-treated cochleae and in the s-EGFP and s-EGFP-9R groups. The scale bar indicates 50 μm. **(b)** Number of SGCs at 28 days. No significant differences in the number of SGCs were found among the treated-side cochleae in the s-EGFP Group, among those in the s-EGFP-9R Group, or among those in the non-treated cochleae. Each *n* = 5.
